# Supplementary material for: Primary motivations for and experiences with paediatric minimally invasive tissue sampling (MITS) participation in Malawi: a qualitative study
Source: BMJ Open. 2022 Jun 8;12(6):e060061. doi: 10.1136/bmjopen-2021-060061 (PMC9185590; doi:10.1136/bmjopen-2021-060061)
Supplement: Supplementary data [file bmjopen-2021-060061supp001.pdf]

Appendix A: Discussion Guide

Number	Topic
T01.	**Introduction**<br>• Offering condolences to the family on the loss<br>• Thanking them for granting Consent to MITS as well as for accepting to be interviewed<br>• Briefing them of their rights (and support available) through the course of the interview<br>• Highlighting the essence of the interview with specific comments on the confidentiality of their responses
T02.	**Socio-demographic details**<br>• Verifying details provided at CHAIN recruitment<br>• Asking on additional information
T03.	**Exit Survey**<br>• Administering exit survey
T04.	**History of illness**<br>• Perceived origins/cause of illness<br>- Traditional causes/Medical causes<br>• Specific stages of illness (e.g. stage that made them decide to go to hospital, inform extended family, who decided on action to be taken etc)<br>• Admission into hospital<br>• Recruitment into the CHAIN study<br>- Consenting for tests while in hospital (including decision making around consent)<br>- Relationship with the main CHAIN team<br>• Previous experience of hospital care<br>• Social support in course of illness
T05.	**Hospital care post death**<br>• Explanation of CoD from the hospital<br>• Understanding of explained CoD<br>- Sufficiency of explained CoD<br>- Alignment with their initial thoughts on CoD<br>• Explained CoD ability to address concerns
T06.	**Approach for MITS**<br>• Consent for MITS<br>• Information given for MITS<br>• Reasons for Consenting to MITS

Lawrence S, *et al. BMJ Open* 2022; 12:e060061. doi: 10.1136/bmjopen-2021-060061

	• Decision making
T07.	**MITS and burial practices**
	• Religious and traditional customs observed in child death and burial<br>- Usual burial rites for child (age specific in relation to tradition/religion)<br>• MITS implication on observing customs
T08.	**Sharing of MITS information**
	• People told about the procedure and reasons for telling them<br>• Reactions of the people told, things heard in hospital or community<br>• Family and HH response to the reactions<br>-
T09.	**Knowledge production**
	• Knowledge gained after participating in MITS<br>• Impact of MITS in relating to hospital/HCWs<br>• Impact of MITS in future home care and care-seeking for illnesses<br>• Overall benefits from the procedure/experience
T10.	**Concluding remarks**
	• Regrets<br>• Recommendations for MITS consent approach<br>• Recommendations on releasing results of MITS to families<br>• Likelihood of recommending MITS<br>• General comments
