# Supplementary material for: Primary motivations for and experiences with paediatric minimally invasive tissue sampling (MITS) participation in Malawi: a qualitative study
Source: BMJ Open. 2022 Jun 8;12(6):e060061. doi: 10.1136/bmjopen-2021-060061 (PMC9185590; doi:10.1136/bmjopen-2021-060061)
Supplement: Supplementary data [file bmjopen-2021-060061supp002.pdf]

Supplemental Table 1. Interview summaries.

Family #	Summary	Primary caregiver(s)	Caregiver(s) religion	Understood diagnosis available	Primary consenter(s)	Interview participant(s)
**1**	Family first went to multiple health centers, where they reported being poorly treated by HCWs. They enrolled in CHAIN in hopes their engagement in the study would improve the child's health. Family very pleased with care by CHAIN team generally. MITS participation was motivated by wanting to better understand child's CoD but did not understand MITS consent procedures well.	Mother and father	Pentecostal	Yes	Mother and father	Mother and father
**2**	Child's mother reported not getting along with the father. She had previously enrolled in malaria research and enrolled the child in CHAIN. Mother was pleased with CHAIN care but displeased with HCWs at night. She was motivated to know her child's CoD, especially if hereditary.	Mother	Pentecostal	No	Mother (grandmother present for support only)	Mother
**3**	Child's mother was displeased with care provided when child's condition was deteriorating. She consented independently to CHAIN but did not participate in the MITS consent process, deferring to tradition. Child's father and grandfather wanted to know CoD, thinking clinical diagnosis might not be the "real" CoD.	Mother	Protestant	Yes	Father and maternal uncle	Mother
**4**	Child was receiving care at a health center for a month before admission to QECH. Child was enrolled in CHAIN. Family was interested in learning CoD and did not want to take the time to solicit input from other family members during consent process.	Mother and father	Pentecostal	No	Father	Mother and father
**5**	Family did not want to seek care at local health center because they believed care quality there to be poor. Child was enrolled in CHAIN. Child's father had trouble visiting during admission because they lacked funds for transport. He was not involved in consent process. Child's maternal aunt was involved in the MITS consent and initially expressed concerns about organ harvesting. The mother wanted to know CoD.	Mother and father	Pentecostal	No	Mother, maternal aunt and cousin, and cousin's husband	Mother

**6**	Child's father left mother after family tested HIV positive and accused mother of sex work. He visited during child's admission at QECH but was not involved in the consent process. Child was enrolled in a nutrition substudy of CHAIN and mother felt supported by HCWs at QECH. Child's mother was interested in better understanding child's CoD but was primarily motivated by transport and coffin provision, as told to her by staff on the wards before the consent process began.	Mother	Catholic	Yes	Mother	Mother
**7**	Child was enrolled in a nutrition substudy of CHAIN. Family was influenced by transport provision in their decision to consent. Mother consented alone before the child's uncle arrived and he confirmed to proceed.	Mother	Catholic	No	Mother and grandmother	Mother, grandmother, and uncle
**8**	Child's mother was young (last year of secondary school) and initially hid her pregnancy. Child's father recently had a stroke and was receiving treatment. Family was very upset with care, especially lack of testing they were told was going to be done. Family's motivation to participate in MITS was to provide the CoD results to the father's family if they asked, but only if they asked. All three participants were from different faith groups (Islam, Protestant, and Catholic) but raised as Muslim.	Mother	Protestant	Yes	Mother, grandmother, and maternal aunt	Mother, grandmother, and maternal aunt
**9**	Child had sustained severe burns that were being managed on an outpatient basis prior to the last hospital admission. The father's family's church prohibits hospital treatment. However, family elects to seek treatment when needed and accepts church reprimands. While the mother was not involved in MITS consent process (she had returned home), she reported she would have consented as well.	Mother and father	Apostolic	Yes	Maternal aunt and her husband, and maternal uncle	Mother and father
**10**	Child's father visited QECH during the hospital stay. Both parents were dissatisfied with the way they were treated at hospital, and they were particularly upset the mother was never asked about child's condition. Father initially consented by phone. He did not seem to have a clear understanding of the MITS procedure. Consent motivated by feeling that caregivers should do as HCWs ask.	Mother and father	Catholic	No	Father	Mother and father

**11**	Family seemed satisfied with communication by HCWs, although limited. Family was keen to know more about CoD because they did not think the child would die as a consequence of pneumonia diagnosis. Child was buried the day after death and family thought they would have received pressure to not consent by the sub-chief if he would have been involved in decision-making.	Mother	Data unavailable	Yes	Father by phone, maternal grandmother, and great aunt	Mother and paternal grandmother
**12 (both twins enrolled in MiM)**	Child's mother was struggling with mental health condition and limited resources during twins' life. She had quit taking antiretroviral medications for HIV infection prior to their deaths at the recommendation of a local "prophet". Twins' father left their mother before their birth. The mother died shortly after twins' deaths and the uncle was interviewed. They had consented to MITS as a family, hoping it would alleviate blame for the twins' mother.	Mother	Apostolic	Data unavailable	Mother, maternal uncle, and maternal grandmother	Uncle
**13**	Child's mother was not satisfied with care received at a nearby public health center and at a private clinic, so she sought care at QECH without a referral. Family was satisfied with the care at QECH. Family consented to MITS to gain CoD information to protect surviving twin.	Mother and father	Pentecostal	No	Mother, father, paternal aunt, and female friend	Mother and father
**14**	Child's mother left child with father at 5 months after he married a second wife. Biological mother stayed with child at QECH, but grandparents (primary caregivers) did not seek mother's input during consent process because she was no longer the guardian. Health treatment was sought at a health center for child's condition prior to admission at QECH. Child was enrolled in a nutrition study. Family consented because they were interested in learning CoD.	Paternal grandparents	Islam	No	Paternal grandparents, (paternal aunt also present)	Paternal grandparents and stepmother
**15**	Child's father visited occasionally during QECH admission. He noted receiving no information from HCWs during visits. Family consented because they were interested in learning CoD and felt it would be helpful to their community, but they had no intention to share the results due to fears of rumors and misconceptions. They were relieved to receive transportation.	Mother and father	Protestant	Data unavailable	Uncle and father	Uncle and father

Abbreviations: CoD, cause of death; HCWs, healthcare workers; QECH, Queen Elizabeth Central Hospital
