# Supplementary material for: Primary motivations for and experiences with paediatric minimally invasive tissue sampling (MITS) participation in Malawi: a qualitative study
Source: BMJ Open. 2022 Jun 8;12(6):e060061. doi: 10.1136/bmjopen-2021-060061 (PMC9185590; doi:10.1136/bmjopen-2021-060061)
Supplement: Author's manuscript [file bmjopen-2021-060061.draft_revisions.pdf]

BMJ Open is committed to open peer review. As part of this commitment we make the peer review history of every article we publish publicly available.

When an article is published we post the peer reviewers' comments and the authors' responses online. We also post the versions of the paper that were used during peer review. These are the versions that the peer review comments apply to.

The versions of the paper that follow are the versions that were submitted during the peer review process. They are not the versions of record or the final published versions. They should not be cited or distributed as the published version of this manuscript.

BMJ Open is an open access journal and the full, final, typeset and author-corrected version of record of the manuscript is available on our site with no access controls, subscription charges or pay-per-view fees (http://bmjopen.bmj.com).

If you have any questions on BMJ Open's open peer review process please email info.bmjopen@bmj.com

BMJ Open

Primary motivations for pediatric minimally invasive tissue sampling (MITS) participation, consent dynamics, and intended use of cause of death results in Malawi: Implications for ethical practice in MITS research

Journal:	*BMJ Open*
Manuscript ID	bmjopen-2021-060061
Article Type:	Original research
Date Submitted by the Author:	13-Dec-2021

Complete List of Authors:	Lawrence, Sarah; University of Washington, Pediatrics<br>Namusanya, Dave; Abertay University, Behaviour and Health Research Group; Malawi-Liverpool-Wellcome Trust Clinical Research Programme, Behavior and Health Research Group<br>Mohamed, Sumaya; University of Washington, Global Health<br>Hamuza, Andrew; Malawi-Liverpool-Wellcome Trust Clinical Research Programme, Behaviour and Health Research Group<br>Huwa, Cornelius; University of Malawi College of Medicine, Paediatrics<br>Chasweka, Dennis; University of Malawi College of Medicine, Paediatrics; The Childhood Acute Illness & Nutrition Network<br>Kelley, M; University of Oxford, Health Systems Collaborative, Nuffield Department of Medicine; The Childhood Acute Illness & Nutrition Network<br>Molyneux, Sassy ; University of Oxford, Health Systems Collaborative, Nuffield Department of Medicine ; The Childhood Acute Illness & Nutrition Network<br>Voskuijl, Wieger ; Amsterdam University Medical Centres, Pediatrics; The Childhood Acute Illness & Nutrition Network<br>Denno, Donna; University of Washington, Department of Pediatrics; The Childhood Acute Illness & Nutrition Network<br>Desmond, Nicola; Liverpool School of Tropical Medicine, International Public Health; Malawi-Liverpool-Wellcome Trust Clinical Research Programme, Behaviour and Health Research Group

Keywords:	PAEDIATRICS, TROPICAL MEDICINE, PUBLIC HEALTH, Anthropology < TROPICAL MEDICINE

SCHOLARONE™
Manuscripts

I, the Submitting Author has the right to grant and does grant on behalf of all authors of the Work (as defined in the below author licence), an exclusive licence and/or a non-exclusive licence for contributions from authors who are: i) UK Crown employees; ii) where BMJ has agreed a CC-BY licence shall apply, and/or iii) in accordance with the terms applicable for US Federal Government officers or employees acting as part of their official duties; on a worldwide, perpetual, irrevocable, royalty-free basis to BMJ Publishing Group Ltd ("BMJ") its licensees and where the relevant Journal is co-owned by BMJ to the co-owners of the Journal, to publish the Work in this journal and any other BMJ products and to exploit all rights, as set out in our licence.

The Submitting Author accepts and understands that any supply made under these terms is made by BMJ to the Submitting Author unless you are acting as an employee on behalf of your employer or a postgraduate student of an affiliated institution which is paying any applicable article publishing charge ("APC") for Open Access articles. Where the Submitting Author wishes to make the Work available on an Open Access basis (and intends to pay the relevant APC), the terms of reuse of such Open Access shall be governed by a Creative Commons licence – details of these licences and which Creative Commons licence will apply to this Work are set out in our licence referred to above.

Other than as permitted in any relevant BMJ Author's Self Archiving Policies, I confirm this Work has not been accepted for publication elsewhere, is not being considered for publication elsewhere and does not duplicate material already published. I confirm all authors consent to publication of this Work and authorise the granting of this licence.

For peer review only

Primary motivations for pediatric minimally invasive tissue sampling (MITS) participation, consent dynamics, and intended use of cause of death results in Malawi: Implications for ethical practice in MITS research

Sarah Lawrence[1†], Dave Namusanya[2,3†], Sumaya B. Mohamed[4], Andrew Hamuza[3], Cornelius Huwa[5,6], Dennis Chasweka[5,6], Maureen Kelley[6,7], Sassy Molyneux[6,7], Wieger Voskuijl[5,6,8,9], Donna M. Denno[1,4,6], Nicola Desmond*[3,10]

† shared first authorship

[1] Department of Pediatrics, University of Washington, Seattle, Washington, United States of America

[2] Behaviour and Health Research Group, Abertay University, Dundee, United Kingdom

[3] Behaviour and Health Research Group, Malawi-Liverpool-Wellcome Trust, Blantyre, Malawi

[4] Department of Global Health, University of Washington, Seattle, Washington, United States of America

[5] Department of Paediatrics and Child Health, Kamuzu University of Health Sciences (formerly College of Medicine), Blantyre, Malawi

[6] The Childhood Acute Illness & Nutrition Network, Nairobi, Kenya

[7] Health Systems Collaborative, Nuffield Department of Medicine, University of Oxford, United Kingdom and KEMRI-Wellcome Trust Research Programme, Kilifi, Kenya

[8] Amsterdam Institute for Global Health and Development, Department of Global Health, Amsterdam University Medical Centres, Amsterdam, The Netherlands

[9] Amsterdam Centre for Global Child Health, Emma Children's hospital, Amsterdam University Medical Centres, Amsterdam, The Netherlands

[10] Department of International Public Health, Liverpool School of Tropical Medicine, Pembroke Place, Liverpool, United Kingdom

***** Corresponding author

Email: nicola.desmond@lstmed.ac.uk (NK)

Word Count: 4901

For peer review only - http://bmjopen.bmj.com/site/about/guidelines.xhtml

ABSTRACT

Introduction: Postmortem minimally invasive tissue sampling (MITS) uses biopsy needles to sample body fluid and tissue for histopathological and microbiologic investigation. It is increasingly being used globally to improve cause of death (CoD) attribution; however, post-procedure assessments of family experiences related to MITS remain limited. We sought to understand family member consent decision-making influences and experiences in Malawi in order to inform future MITS studies.

Methods: We conducted in-depth interviews with 16 families six weeks after the death of pediatric MITS participants and analyzed the data using a combination of thematic content and framework approaches.

Results: Improved CoD ascertainment was the principal motivator for participation to protect remaining or future children. Community burial norms, religious doctrine, and relationships with healthcare workers were not reported influencers among family members who consented to the procedure. Primary consenters varied, with single mothers more likely to consent independently or with only female family members present. Clear understanding of MITS procedures appeared limited six weeks post-procedure, but research was described as voluntary and pre-consent information satisfactory for decision-making. Most families intended to share about MITS only to those involved in the consent process, for fear of rumors or judgment by extended family members and the wider community.

For peer review only - http://bmjopen.bmj.com/site/about/guidelines.xhtml

Conclusion: Among those who consented to MITS, decision-making was informed by individual and household experiences and beliefs, but not by religious affiliation or experiences with HCWs. While understanding of the MITS procedure was limited at the time of interview, families found informed consent information sufficient for decision-making. Future MITS studies should continue to explore information presentation best practices to facilitate informed consent during the immediate grieving period.

ARTICLE SUMMARY

Strengths and limitations of this study

- This study conducted in-depth interviews with families following MITS participation to improve understanding of MITS decision-making factors, experiences with the consent process, and plans for MITS results utilization.
- The MiM study was pediatric and hospital-based, therefore findings may not be generalizable to adult or community-based MITS studies or to settings outside of southern Malawi.
- We lack in-depth data from families who declined to consent to MITS, hence our detailed findings only reflect the views shared by family members who consented to the study.

INTRODUCTION

Improved cause of death (CoD) determination is essential to tailor intervention development and implementation to reduce preventable child deaths. Complete diagnostic autopsy (CDA) is the gold

For peer review only - http://bmjopen.bmj.com/site/about/guidelines.xhtml

For peer review only

standard for determining CoD but is often challenging or unfeasible due to resource constraints, limited acceptability linked to cultural and social norms, and concerns about body disfigurement. In response to these barriers, minimally invasive tissue sampling (MITS) has been validated and is increasingly being utilized as an alternative to CDA to establish CoD in various settings, including among child deaths.[1, 2] MITS uses small needles to sample organs and body fluids for histopathological and microbiologic investigations to inform CoD determinations.[3]

Due to the sensitivity of the procedure, the immediate personal tragedy faced by parents/caregivers from whom consent is sought, and the need to evaluate context-specific acceptability, pre-implementation formative acceptability research has been widely implemented.[4-11] MITS has generally been found to be more acceptable than CDA across diverse settings, reducing some of the cultural, social, and structural restrictions that constrain CDA implementation.[4-6, 12, 13] In particular, body disfigurement—a commonly cited reason for declining CDA – is mitigated.[4-6, 13] Formative research to understand *hypothetical* pediatric MITS acceptability in Malawi, found that acceptability would likely be strongly influenced by social relationships between parents, extended family, hospital workers, and research staff, but that religious affiliation would be less influential than individual and household beliefs and practices.[14] Participants believed MITS participation would be facilitated by desire for improved CoD information but hindered by fears of organ harvesting and disruption to transportation and burial plans, corroborating evidence from similar studies in other settings.[10, 15] Body disfigurement was still raised as a concern in the Malawi formative assessment.[14]

While evidence on MITS acceptability derived from pre-implementation assessments has increased in recent years, post-participation assessments have been limited.[8] With increasing MITS utilization globally, it is important to assess how families perceive the procedures after participation to optimize

For peer review only - http://bmjopen.bmj.com/site/about/guidelines.xhtml

consent and support processes for grieving families. This study aims to better understand decision-making factors and experiences among family members who consented to a pediatric MITS study in Malawi to optimize future MITS studies.

METHODS

Study setting

The MITS in Malawi (MiM) study was conducted at Queen Elizabeth Central Hospital (QECH) which serves as the central referral hospital for southern Malawi. Services are largely provided free of charge. Eligibility included children (7 days to 4 years) who died during hospitalization with acute illness and/or malnutrition and with a primary caregiver residing within a 50-kilometer radius of QECH. This catchment area includes ethnically diverse, predominantly Christian, matrilocal urban and rural communities.

MiM started as a sub-study of the Childhood Acute Illness & Nutrition (CHAIN) Network, which aims to identify mortality risk factors among children hospitalized with acute illness or undernutrition across nine sites in six countries,[16] but was expanded to patients enrolled in two other undernutrition studies conducted by the same research group and to the general pediatric wards. Caregivers approached for enrollment were offered transportation and a coffin after information about the study was provided, irrespective of participation decision.

Study design and sampling

In-depth interviews were conducted with family members who consented to the MITS procedure to understand experiences with the consent process, decision-making factors, and their plans for using and

For peer review only - http://bmjopen.bmj.com/site/about/guidelines.xhtml

sharing of MITS results. During the consent for MITS, written consent was obtained from the family for interviews six weeks later at QECH or at their home, whichever they preferred. Transport for participants to and from the interview was provided by the study. Those who did not consent to MITS were asked their reasons for withholding consent.

A discussion guide (Appendix A) was developed in English, based on formative qualitative research exploring MITS acceptability in Malawi.[14] It was translated to Chichewa (the local language). Sociodemographic data was available from linked case report forms for participants co-enrolled in other studies and additional sociodemographic data was collected during the interview. Interviews focused on experiences within the hospital prior to death, interactions with the MITS study team, consent procedures, motivations for participation, and intended use of MITS results.

Data collection and analysis

Interviews were conducted between September 2018 and December 2019. All participants preferred to receive MITS results and be interviewed at QECH. Participants first met with the study team to receive CoD results. They then provided verbal consent to be interviewed. Interviews were conducted by a male, masters-level social scientist (DN, shadowed by AH) who had not previously met the participants and was introduced by the clinical MITS study team member who provided CoD results and answered any questions about the findings. Interviews were conducted in Chichewa largely using a narrative approach led by participants while addressing discussion guide topics. Interviews were recorded, transcribed, and translated verbatim. Post-interview reflections (by DN) were noted to capture key topics and initial thoughts.

For peer review only - http://bmjopen.bmj.com/site/about/guidelines.xhtml

Transcripts were analyzed using a codebook that was developed iteratively through reading post-interview reflection reports and transcripts, code development and refinement, preliminary code application to transcripts, discussion of code application, and revision to the codebook. Transcripts were imported into NVivo 11 Plus (QSR International Pty Ltd). Each transcript was coded independently by one team member (DN, SL, SBM). Transcripts were exchanged and coded transcripts were reviewed by a second coder. Coding disagreements were resolved through group discussion.

Interviews were analyzed using thematic content and framework analysis to produce a description of key themes across all interviews.[17, 18] Coded data were grouped into themes and then explored by socio-demographic features, such as primary caregiver, past experiences in research, and religion to identify if key characteristics influenced MITS decisions, participation experiences, and reflections following CoD results provision. Individual case study descriptions were also produced to explore situated experiences in-depth, drawing on a phenomenological approach that recognizes the unique experiences of individuals, especially where there is little prior knowledge available about the topic.[19]

Ethics approvals

The Malawi National Health Sciences Research Committee (Protocol #17/09/1913) and the Oxford Tropical Research Ethics Committee (Reference 34-16) approved the study protocols. The University of Washington Institutional Review Board (STUDY00003689) exempted the study from review. Written informed consent was provided by all participants.

Patient and public involvement

Patients or the public were not involved in the design, or conduct, or reporting, or dissemination plans of our research.

For peer review only - http://bmjopen.bmj.com/site/about/guidelines.xhtml

RESULTS

Of the 58 families approached for participation in MiM, 29 (50%) consented (Figure 1). Families most often cited lack of benefits as the primary refusal reason – if they were already satisfied with the CoD information provided – followed by not wanted to delay burial, and religious concerns including beliefs that death is God's will and therefore needs no further investigation or that postmortem procedures are explicitly prohibited by their specific place of worship. Two families were disappointed in the care their child received during hospitalization and felt more should have been done while the child was alive, not research after death. Two other families were unable to reach the family member with the authority to consent (as defined by the family – father and uncle for one case and biological mother for another) and another family felt their child was too young (2 weeks old) to have postmortem procedures conducted.

Figure 1. Consent to MITS and interviews.

Of the 29 families who consented to the MITS procedures, 16 participated in interviews. Nine families were not approached for interview due to social scientist unavailability (cases 21-29) and three could not be reached for interview and results provision. 16 of 16 families approached for interviews consented. One interview was omitted due to poor audio quality. Interviews included one to three family members, most often the mother and father together, followed by the mother alone (Table 1). Interviews lasted between 31 and 91 minutes.

Table 1. Characteristics of participants (n=15).

Characteristic	Median (IQR) or n (%)
Relationship to MITS participant	
Mother & father	5 (33.3)
Mother	4 (26.7)
Mother, grandmother, & aunt/uncle	3 (20.0)

For peer review only
For peer review only - http://bmjopen.bmj.com/site/about/guidelines.xhtml

Father & uncle	1 (6.7)
Uncle	1 (6.7)
Stepmother & grandparents	1 (6.7)
Prior child death in family	
Yes	1 (12.5)
No	4 (26.7)
N/A - first child	4 (26.7)
Data unavailable*	6 (40.0)
Primary caregiver's religion	
Pentecostal	5 (33.3)
Catholic	3 (20.0)
Protestant	3 (20.0)
Apostolic	2 (13.3)
Islam	1 (6.7)
Data unavailable*	1 (6.7)
Prior research participation	
CHAIN or other nutrition study	8 (53.3)
Other research prior to admission	1 (6.7)
Data unavailable*	6 (40.0)
Reported informed of child's diagnosis during life	
Yes	6 (40.0)
No	7 (46.7)
Data unavailable*	2 (13.3)
**Characteristics of MITS participant**	
Female	4 (26.7)
Age (months)	7.6 (3.0, 16.3)
Living twin	2 (12.5)
Primary caregiver	
Mother	6 (40.0)
Mother & father	8 (53.3)
Grandparents	1 (6.7)
Number of living biological siblings	
3+	5 (33.3)
2	2 (13.3)
1	1 (6.7)
0	6 (40.0)
Data unavailable*	1 (6.7)
Inpatient stay duration (days)	4.0 (2.0, 8.0)

*not discussed in interview with family

Definitions: MITS, minimally invasive tissue sampling; N/A, not applicable

Participant characteristics of each are summarized in Supplemental Table 1, including reasons for participating in MITS and consent dynamics. Socio-demographic characteristics, such as primary

For peer review only - http://bmjopen.bmj.com/site/about/guidelines.xhtml

caregiver and religion, were unrelated to factors described as influencing decision-making, amongst those who participated.

For those who consented, MITS participation was largely reported to be influenced by desire for improved CoD knowledge, individual beliefs about burial traditions and religious practices, and in some instances, transportation and coffin provision. Participants reported that interactions with health care workers (HCWs) or researchers, either positive or negative, did not influence their decision-making. Primary consenters were identified by families and varied depending on each particular situation. Clear understanding of the MITS procedure was often limited at the time of interview. Most participants did not intend to share MITS results outside of those involved in the consent process. The strength of themes varied across interviews (Figure 2).

Figure 2. Strength of themes by family.

Reasons for MITS research participation

Knowledge-seeking

Desire for improved CoD information was the primary driver of MITS participation. Caregivers saw MITS as an opportunity to learn about the cause of illness and subsequent death, especially when a clear, well-understood diagnosis was not reported as provided during the child's life. Most families provided with diagnoses during the child's life believed the information was insufficient, due to limited information provided by HCWs or their own limited understanding. They often believed this prior information was not representative of the "true" CoD.

For peer review only

For peer review only - http://bmjopen.bmj.com/site/about/guidelines.xhtml

…if we refused [MITS], we would not have known the cause of the death because we were just thinking that it's just mere diarrhea. We didn't really know the disease, so I wanted to know the cause of death. (Mother, Family 2)

For most families, CoD information-seeking was driven by a desire to protect remaining or future children through identification of hereditary illnesses or insights that might guide child-rearing practices. Two families reported motivation to protect the life of the living twin, both of whom also had two or more other living siblings.

We were grappling with questions as parents and we were wondering that with this sort of death, then how best can we take care of the surviving twin child. So we said, let us consent and then they can find out the actual cause of death and if it will help others then also it will help us as parents. (Father, Family 1)

Five participants also identified MITS as beneficial to the wider community through its potential to inform the development of future treatments so others will not have to suffer the loss of a child.

We agreed to do postmortem so that the findings could help someone in future. We don't want what happened to us to happen to other people. (Father, Family 13)

Individual beliefs about burial and religion

Participants shared that their decision to participate was strongly influenced by individual beliefs about death and religion rather than by broader community burial norms or religious doctrine.

For peer review only - http://bmjopen.bmj.com/site/about/guidelines.xhtml

Burial

Participants reported they were not concerned with potential burial delays or morticians cleaning the body, instead of following traditional burial practices whereby the family cleans the body prior to burial. Practices regarding burial traditions were seen as guidelines, more than rules.

> *We are used to washing the body before burial so when we arrived at the village and the people asked about washing the body, we told them that the body had already been washed and they said that it is not a problem.* (Mother, Family 1)

Some families whose child died in the late afternoon elected to wait for the MITS procedure to be conducted the next morning, even though burials are usually conducted on the same day. These participants were particularly motivated to learn CoD information that they hoped MITS would provide and felt that travel by night would be logistically difficult. They did not view waiting for MITS as a significant breach of tradition. Although, ensuring prompt burial was the primary reason stated for refusing participation in MITS for some families (Fig 1).

Religion

Interviewees viewed MITS participation benefits as outweighing community expectations and norms defined by particular religious affiliation. For example, one family with membership in an Apostolic church whose members usually oppose biomedical health care chose to both seek inpatient and outpatient medical treatment when their child was severely burned, but to also participate in MITS to learn more about the child's death.

For peer review only - http://bmjopen.bmj.com/site/about/guidelines.xhtml

They [church leaders] say that do not go to the hospital but it's up to you the patient to decide based on how you are feeling…you just discipline yourself [after going to the hospital]. (Mother, Family 10)

Religious affiliations were not known for all of the five families who declined MITS and cited religious or cultural reasons as their primary concern, although one, for example, noted their Presbyterian church does not allow procedures on the deceased. Another noted they do not believe further investigation is needed after death because death is the will of God.

Relationships with HCWs

Communication with HCWs and research staff, regardless of whether deemed to be satisfactory, did not emerge as a primary factor in consent decision-making for participants. They had variable experiences with HCWs during the QECH inpatient stay and at prior health facilities. Most participants noted some rudeness, limited communication, or lack of follow through by HCWs at some point in their child's care but were ultimately motivated by improved CoD understanding. Participants whose child was enrolled in research studies during life mentioned that researchers exhibited more compassion and attentiveness toward them than clinical staff on the wards.

When I came to the hospital and met with doctors from CHAIN, I could see that they would talk to me with respect; yet when I met with the others, the doctors from the wards, they just spoke to me rudely. They just spoke as if they are tired with their job. (Father, Family 1)

For peer review only - http://bmjopen.bmj.com/site/about/guidelines.xhtml

Participants acknowledged they did not usually ask questions of HCWs on the wards, rather they merely observed, because HCWs have authority over information regarding their child's treatment.

> *They did not tell us [about the child's treatment], we were just watching being that they are the doctors.* (Grandmother, Family 8)

A few of the participants shared they were counseled about their child's deteriorating health and imminent death, although others voiced concerns that HCWs had not responded to their child's deterioration.

While families had mixed experiences with HCWs prior to the child's death, none identified this as a primary factor in deciding to consent to MITS. However, two families declined MITS due to frustration with the care provided during the child's life.

> *Nobody came, they were just busy playing with their phones, but they saw that I was stressed up with the child, they were just looking at him, maybe they knew that the child was dying….* (Mother, Family 10)

Transportation and coffin provision

HCWs involved in care during the inpatient stay introduced the family to MiM research team. A few families noted they were advised by HCWs that the MiM study presented an opportunity for transportation and a coffin, and this strongly influenced their consent decision-making.

For peer review only - http://bmjopen.bmj.com/site/about/guidelines.xhtml

What made me to accept was that I wanted to know the cause of the death because he was having diarrhea but also, I wanted to be helped with transport to get home. (Mother, Family 6)

When the research team became aware of this practice by some HCWs partway through the study, a hospital staff re-training was implemented to reinforce that transport and coffins would be provided regardless of consent and offered to the families by the research team after approach for consent was complete. The finding of this influencer was no longer observed in interviews conducted subsequent to the re-training (post Family 11).

MITS consent dynamics

Primary consenters

Household norms and familial support in the hospital informed who was involved in the MITS consent process and who retained the ultimate authority to consent or not. Most primary caregivers consulted other family members during the decision-making, often including the child's grandparents, uncles, and aunts – especially when they lived nearby or were involved in the child's care during life. When multiple family members were involved, the primary decision-maker for MITS often was the male head of household or the child's maternal uncle.

I could not give consent on this on my own…we [researcher and grandmother] left together with my aunt and sat beside the mortuary, we [grandparents] linked up. After linking up, it was found that the dad [child's grandfather] agreed and said he had given consent on this. (Grandmother, Family 14)

For peer review only - http://bmjopen.bmj.com/site/about/guidelines.xhtml

Some parents did not consult with extended family members, as they believed they held the sole responsibility of their child, including after death. Place of residence (rural vs. peri-urban) did not influence the primary consenter choice within the family.

> *We did not go to consult because we are the parents, the responsibility towards the child rests with us; that is why we just accepted that they should go ahead because we also wanted to know what the problem was.* (Father, Family 1)

Mothers were more likely to consent alone if the father was uninvolved in the child's care generally or if there were marital difficulties.

> *I did everything on my own. There was nobody to discuss with. I was like a father and a mother, so I was supposed to make decisions on everything.* (Mother, Family 2)

Single mothers were also more likely to only engage women in the consent process, often their mothers and sisters who were supporting them during the child's hospital stay or first to arrive. One woman who had a strained relationship with the child's father consented with the child's maternal grandmother present, who had been with her during the child's hospitalization, and then later informed the father of the decision, but she did not feel he needed to be involved in the process.

Interpretation and understanding

For peer review only - http://bmjopen.bmj.com/site/about/guidelines.xhtml

Interviewees were often unable to describe the MITS procedure. The only component one participant recalled (erroneously) was the removal of the brain, noting that this was necessary to determine the CoD. She reported satisfaction with the information provided during the consent process but could not recall further details. Another participant described the procedure as an "x-ray". Two participants highlighted MITS would test more than one area of the body–one noted three to four areas would be tested, while another that "each and every part of the body" would be tested. Only two participants explicitly noted that MITS involves needle sampling and no incisions.

> *They explained to me saying: if possible, there is need to examine the bodies of the children, we will not tear the bodies in any way, but we just use some needles. So that, once we find out about something in the future, since we still have a generation, maybe this can be something to say: we should get prepared now, before something happens. Therefore, according to the way they explained, I felt they gave in some good points.* (Uncle, Family 12)

> *The researchers said their research is not about cutting the body anywhere. They said they will use an injection to pull out fluid from the nerves or from the spinal cord. They said they will get these samples from 3 or 4 areas. When they get these samples, they will tell us the results after one month. I told them, there is no problem. They said after taking the samples, we will ask you to go and confirm that we have not cut anywhere.* (Father, Family 13)

Across all the participants who described the consent procedures, they understood MITS aimed to provide additional information about the child's CoD and their participation was not mandatory.

For peer review only - http://bmjopen.bmj.com/site/about/guidelines.xhtml

So the hospital people came saying that if the death happening like this [you can participate in MITS] but it's not a compulsory thing. We take the body to the mortuary for MITS to find out the real cause of the death. (Mother, Family 8)

Sharing MITS results

Participants reported being satisfied with the CoD results received from the MITS research team and were able to explain the findings and recommendations provided. While many participants who consented to MITS identified better understanding of their child's CoD as protective for their households and communities, very few of them planned to share information about their participation or results with extended family, neighbors, or other community members. Individuals involved in the consent process were frequently the only individuals with whom parents and caregivers planned to share the results. Participants shared this decision was influenced by fear of what people might say about the procedures and the family themselves.

Somebody who may not be close, once you tell them, instead of them to understand, they begin asking you questions (Grandmother, Family 14). *And they can even tell you that you could be the one responsible for my child's condition, nowadays.* (Stepmother, Family 14)

One participant noted that by telling even one relative, the family risked having their decision shared across the community when that relative tells others. Due to concerns that this potential for gossip would cause issues for the family, he decided not to share about MITS.

For peer review only - http://bmjopen.bmj.com/site/about/guidelines.xhtml

A couple of participants shared they would not have told their family members about MITS had they not already been present at the hospital. Some participants reported telling extended family they had enrolled in a study that was providing transport but did not elaborate further on research activities to avoid questions or judgement. Relatives and community members did not push for additional details because transportation for deceased is often a challenge and this answer avoided what participants considered unwelcome questions.

Only four families informed uncles, traditionally regarded as heads of the extended family, that MITS had been conducted.

> *I will tell members of my extended family since they are my nephews and nieces…so they should know what caused the child's death.* (Father, Family 10)

The four families also reported they intend to share information learned about the CoD with these uncles. Of the four, one participant was primarily motivated to share results to prevent blame within the family.

> *People should be able to understand to say: it happened this way, the main issue was that this person died of this problem, so that there should not be finger pointing towards one another.* (Uncle, Family 12)

DISCUSSION

For peer review only - http://bmjopen.bmj.com/site/about/guidelines.xhtml

Our findings demonstrate that individual experiences and familial relationships are central to MITS consent and participation processes. Socio-demographic characteristics did not clearly influence decision-making themes, consent processes, or intention to share results. One notable exception was that single mothers were more likely to consent to MITS independently or with only female family members present, possibly explained by the matrilocal setting.[20] In cases where the father was at least somewhat involved, they were engaged in the consent process and often had the principal authority to consent, corroborating hypothetical findings from formative MITS acceptability research in Mozambique, South Africa, and Pakistan.[6, 8, 9] Interestingly, relationships with HCWs or research staff (neither positive nor negative) or dissatisfaction with the healthcare provided, did not seem to strongly influence decision-making among MiM participants, even though many did report negative interactions. This is in contrast to qualitative work pre-MiM study implementation from our group and other formative studies that suggested trust in the healthcare system and relationships with HCWs and research staff would be important drivers of consent.[14] However, the data from our in-depth interviews are only among those who *consented to MITS* and reflect families' experiences six weeks after the death and MITS procedure.[4, 5, 14] Nevertheless, for these families, it was apparent that desire for improved CoD knowledge outweighed any negative experiences during the hospital stay and participants seemed to accept limited communication with HCWs as the norm. Participants who had received a clear diagnosis of the child's illness prior to death continued seeking additional information after death in a continuous search for meaning, reflecting similar qualitative findings in Tanzania where families continue negotiation in defining illness after death.[21]

While participants often identified MITS results as beneficial to surviving children and potentially protective to the broader community, they frequently did not intend to share their participation experiences or results outside of close family members involved in the consent process. Families were

For peer review only - http://bmjopen.bmj.com/site/about/guidelines.xhtml

fearful of being asked unwanted questions, rumors about their family, and judgment about their participation. This reflects similar findings to formative MITS research in Pakistan that found women fear unexpected CoD results or that new, stigmatized diagnoses would result in blame toward them and the possibility of abandonment by their husbands.[9] MITS researchers need to be acutely aware of inadvertent social harms that CoD results might bring to family members, particularly mothers. Similar to HIV research, MITS investigators should prioritize risk/benefit analyses of MITS participation to promote research participant welfare.[22, 23] As MITS becomes more widely used, experiences of families who have consented to MITS will potentially contribute to wider community acceptability. If families are unable to share their experiences due to fear of social harms, this could negatively influence MITS uptake and normalization of the procedure.

One concerning finding was that some participants were informed, prior to consent approach, by HCWs outside of the research team, of transportation and coffin provision through the MiM study, raising concerns of tacit coercion, albeit driven by in-hospital rumor rather than direct and purposeful communication from study staff. Non-study HCWs presented the study as an opportunity for families with limited resources to receive assistance–trying to help families in their time of need while going against the research protocol which outlined offering transportation and coffin after the approach for consent and regardless of the consent decision. Re-training of HCWs regarding the MiM consent protocol appears to successfully mitigate this practice. Research participation in resource limited settings is often influenced by perceived and actual benefits.[24] Transportation is a much-needed service in Malawi, especially after death because public transit will not transport a body, resulting in transport provision alone to be a sufficient explanation for research participation. This raises ethical concerns around undue inducement, while also highlighting the acute need for support and resources after losing a loved one.[25, 26] However, fear of undue inducement can lead to under-compensation or

For peer review only - http://bmjopen.bmj.com/site/about/guidelines.xhtml

failure to offer essential support for families facing significant difficulties. As such, future MITS studies should carefully consider the benefits provided to all families (regardless of participation) to minimize undue inducement and also support families in need. Researchers in general should carefully monitor how potential participants are introduced to the study team and whether benefits are presented in advance of the informed consent procedures by non-researchers, especially if compensation is only provided for those that consent.

Participants' limited ability to describe the MITS procedures six weeks later also raises questions about the ability to process information soon after the loss of a child and to revisit that information later. One study examining five other MITS sites in sub-Saharan Africa and South Asia reported emotional turmoil after loss of a loved one associated with limited understanding of the procedure during the consent process, requiring additional time for deliberation to consent.[27] While interviewees in this study reported understanding participation as voluntary, they focused more on the benefits and potential outcome of the procedures (improved CoD determination) rather than the procedure itself. Prior to study implementation, formative research was conducted through focus group discussions with HCWs, researchers, community leaders, and parents as well as discussions between researchers to establish how to best present the procedures – including the level of detail – to families.[14] However, interview findings demonstrated that understanding was still limited, but participants generally perceived their understanding of information about the MITS procedure during informed consent processes as satisfactory. Other research studies conducted with bereaved parents in Australia and Canada have found that the majority of individuals feel comfortable making informed consent decisions soon after the loss of a child.[28, 29]

For peer review only - http://bmjopen.bmj.com/site/about/guidelines.xhtml

This study has several limitations, including that we were only able to approach the first two-thirds of families for interview participation and the loss of interview data for one participant due to poor audio recording quality. However, 100% of families approached did participate in post-procedure interviews. The MiM study was pediatric and hospital-based, hence findings may not be generalizable to adult or community-based MITS studies or to settings outside of southern Malawi. Most importantly, we lack in-depth data from families who declined to consent to MITS, hence our detailed findings only reflect the views shared by family members who consented to the study.

Despite these limitations, this study provides an improved understanding of MITS decision-making factors, experiences with the consent process, and plans for MITS results utilization by family members. The importance of improved CoD determination was highlighted as an important benefit of MITS that supersedes some potential barriers to participation, including negative interactions and poor communication with HCWs. This study highlights the importance of monitoring MITS and other studies for unintended undue inducement while also providing adequate support for grieving families with limited financial resources. The study also raises important questions around best practices for information provision in MITS studies to balance sufficient detail for informed consent during an emotionally turbulent period when such content may be less easily absorbed and/or remembered. As MITS studies become more prevalent, the ethical issues raised in this study should continue to be explored and delineated, including across different geographic and cultural contexts.

FUNDING STATEMENT

For peer review only

For peer review only - http://bmjopen.bmj.com/site/about/guidelines.xhtml

This study was funded by the Bill & Melinda Gates Foundation (https://www.gatesfoundation.org/) as a sub-study, led by DD and WV, of the CHAIN Network (grant number OPP1131320). Additional funding for MK and SM provided by a Wellcome Trust and MRC Newton Fund Collaborative Award (https://mrc.ukri.org/news/browse/wellcome-trust-mrc-newton-fund-collaboration/)(grant number 200344). The funders had no role in study design, data collection and analysis, decision to publish, or preparation of the manuscript.

For peer review only

COMPETING INTERESTS

None declared.

DATA AVAILABILITY

The data that support the findings of this study are available on request from the corresponding author, ND. The data are not publicly available due to their containing information that could compromise the privacy of research participants.

ACKNOWLEDGEMENTS

We would like to thank participants for sharing their thoughts and experiences, particularly during a grief-marked period. We would also like to thank Hendrina Kaliati of the Malawi Liverpool Wellcome Trust Clinical Research Programme for transcription and translation of recorded data. We thank the

For peer review only - http://bmjopen.bmj.com/site/about/guidelines.xhtml

Malawi CHAIN team and the CHAIN Network Principal Investigators, Prof. Judd L Walson and Prof. James A Berkley for their support.

AUTHOR CONTRIBUTIONS

ND, DD, WV, SM, MK, and CH conceived of the initial study design. DN, AH, and DC were involved in data collection and data processing. DN and SL managed the data. SL, DN, and SBM contributed to data analysis. SL, DN, ND, and DD led the writing of the paper. All authors read and approved the final manuscript.

REFERENCES

1. Bassat Q, Castillo P, Martinez MJ, et al. Validity of a minimally invasive autopsy tool for cause of death determination in pediatric deaths in Mozambique: An observational study. *PLoS Med* 2017;14(6):e1002317. doi: 10.1371/journal.pmed.1002317.

2. Paganelli CR, Goco NJ, McClure EM, et al. The evolution of minimally invasive tissue sampling in postmortem examination: a narrative review. *Glob Health Action* 2020;13(1):1792682. doi: 10.1080/16549716.2020.1792682.

3. Castillo P, Ussene E, Ismail MR, et al. Pathological methods applied to the investigation of causes of death in developing countries: minimally invasive autopsy approach. *PLoS One* 2015;10(6):e0132057. doi: 10.1371/journal.pone0132057.

4. Maixenchs M, Anselmo R, Zielinski-Gutiérrez E, et al. Willingness to Know the Cause of Death and Hypothetical Acceptability of the Minimally Invasive Autopsy in Six Diverse African

For peer review only

For peer review only - http://bmjopen.bmj.com/site/about/guidelines.xhtml

and Asian Settings: A Mixed Methods Socio-Behavioural Study. *PLoS Med* 2016;13(11):e1002172. doi: 10.1371/journal.pmed.1002172.

5. Gurley ES, Parveen S, Islam MS, et al. Family and community concerns about post-mortem needle biopsies in a Muslim society. *BMC Med Ethics* 2011;12(1):10. doi: 10.1186/1472-6939-12-10.

6. Maixenchs M, Anselmo R, Sanz A, et al. Healthcare providers' views and perceptions on post-mortem procedures for cause of death determination in Southern Mozambique. *PLoS Med* 2018;13(7) doi: 10.1371/journal.pone.0200058.

7. Blevins J, O'Mara Sage E, Kone A, et al. Using Participatory Workshops to Assess Alignment or Tension in the Community for Minimally Invasive Tissue Sampling Prior to Start of Child Mortality Surveillance: Lessons from 5 Sites Across the CHAMPS Network. *Clin Infect Dis* 2019;69(Suppl 4). doi: 10.1093/cid/ciz563.

8. Ngwenya N, Coplan D, Nzenze S, et al. Community Acceptability of Minimally Invasive Autopsy (MIA) in Children Under Five Years of Age in Soweto, South Africa. *Anthropology Southern Africa* 2017;40(2):108-21. doi: 10.1080/23323256.2017.1348237.

9. Feroz A, Ali AS, Ibrahim MN, et al. Perceptions of health professionals regarding minimally invasive tissue sampling (MITS) to identify the cause of death in stillbirths and neonates: results from a qualitative study. *Matern Health Neonatol Perinatol* 2019;5:17. doi: 10.1186/s40748-019-0112-x [published Online First: 2019/11/02]

10. Feroz A, Ibrahim M, McClure E, et al. Perceptions of parents and religious leaders regarding minimal invasive tissue sampling to identify the cause of death in stillbirths and

For peer review only

neonates: results from a qualitative study. *Reprod Health* 2019;10(16):53. doi: 10.1186/s12978-019-0730-9

11. Das MK, Arora NK, Rasally R, et al. Exploring family, community, and healthcare provider perceptions and acceptability for minimal invasive tissue sampling to identify the cause of death in under-five deaths and stillbirths in North India: a qualitative study protocol. *Reprod Health* 2019;16(1):3. doi: 10.1186/s12978-019-0665-1.

12. Ben-Sasi K, Chitty LS , Franck LS, et al. Acceptability of a minimally invasive perinatal/paediatric autopsy: healthcare professionals' views and implications for practice. *Prenatal Diagnosis* 2013;33:307–12. doi: 10.1002/pd.4077

13. Kang X, Cos T Fau - Guizani M, Guizani M Fau - Cannie MM, et al. Parental acceptance of minimally invasive fetal and neonatal autopsy compared with conventional autopsy. *Prenat Diag* 2014;34(11):1106-10. doi: 10.1002/pd

14. Lawrence S, Namusanya D, Hamuza A, et al. Hypothetical acceptability of hospital-based post-mortem pediatric minimally invasive tissue sampling in Malawi: The role of complex social relationships. *PLoS One* 2021;16(2) doi: 10.1371/journal.pone.0246369.

15. Bassat Q, Ordi J, Vila J, et al. Development of a post-mortem procedure to reduce the uncertainty regarding causes of death in developing countries. *Lancet Glob Health* 2013;1(3):e125-e26. doi: 10.1016/S2214-109X(13)70037-8.

16. Berkley JA, Walson JL, Diallo AH, et al. Childhood Acute Illness and Nutrition (CHAIN) Network: A protocol for a multi-site prospective cohort study to identify modifiable risk factors for mortality among acutely ill children in Africa and Asia. *BMJ Open*

For peer review only

For peer review only - http://bmjopen.bmj.com/site/about/guidelines.xhtml

2019;9(5):e028454. PMID:31061058; PMCID:PMC6502050. doi:10.1136/bmjopen-2018-028454

17. Hsieh H-F, Shannon SE. Three approaches to qualitative content analysis. *Qual Health Res* 2005;15(9) doi: 10.1177/1049732305276687

18. Boeije HR. A purposeful approach to the constant comparative method in the analysis of qualitative interviews. *Quality & Quantity* 2002;36(4):391-209.

19. Reeves S, Albert M, Kuper A, et al. Why use theories in qualitative research? *BMJ* 2008;337(a949) doi: https://doi-org.offcampus.lib.washington.edu/10.1136/bmj.a949

20. Ewing V, Tolhurst R, Kapinda A, et al. Increasing understanding of the relationship between geographic access and gendered decision-making power for treatment-seeking for febrile children in the Chikwawa district of Malawi. *Malar J* 2016;15 doi: 10.1186/s12936-016-1559-0

21. Desmond N, Prost A, Wight D. Managing risk through treatment-seeking in rural north-western Tanzania: Categorising health problems as malaria and *nzoka*. *Health, Risk, & Society* 2012;14(2):149-70. doi: 10.1080/13698575.2012.661042

22. Sookan T, Zihindula G, Wassenaar D. Standard of care for social harms in HIV prevention trials: A South African perspective. *Developing World Bioeth* 2019;20:194-99. doi: 10.1111/dewb.12255

23. Montgomery ET, Roberts ST, Nel A, et al. Social harms in female-initiated HIV prevention medical research state of the evidence. *AIDS* 2019;33(14):2237-44. doi: 10.1097/QAD.0000000000002346

For peer review only

For peer review only - http://bmjopen.bmj.com/site/about/guidelines.xhtml

24. Mfutso-Bengo J, Ndebele P, Jumbe V, et al. Why do individuals agree to enrol in clinical trials? A qualitative study of health research participation in Blantyre, Malawi. *Malawi Med J* 2008;20(2):37-41. doi: 10.4314/mmj.v20i2.10898.

25. Klitzman R. The Importance of Social, Cultural, and Economic Contexts, and Empirical Research in Examining "Undue Inducement". *Am J Bioethics* 2005;5(5):19-21. doi: 10.1080/15265160500245014

26. Hyun I. Fair payment or undue inducement? *Nature* 2006;442:629-30. doi: 10.1038/442629a

27. Feroz A, Paganelli C, Bunei M, et al. A comparison of MITS counseling and informed consent processes in Pakistan, India, Bangladesh, Kenya, and Ethiopia. *Reproductive Health* 2020;17(120) doi: 10.1186/s12978-020-00969-w

28. Beck AM, Konner C, A. Ethical issues in the study of bereavement: The opinions of bereaved adults. *Death Studies* 2007;31:783-99. doi: 10.1080/07481180701537220

29. Whitfield V, Havyatt J, Buckley T, et al. The complexities of recruiting bereaved family members into a research study in the critical care environment: A discussion paper. *Australian Critical Care* 2015;28:77-81.

For peer review only

For peer review only - http://bmjopen.bmj.com/site/about/guidelines.xhtml

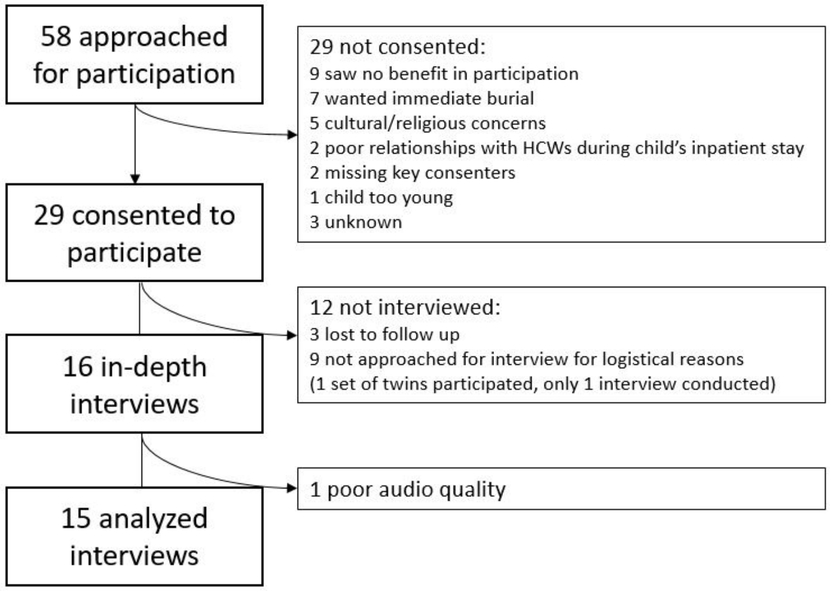

Figure 1. Consent to MITS and interviews.

235x160mm (96 x 96 DPI)

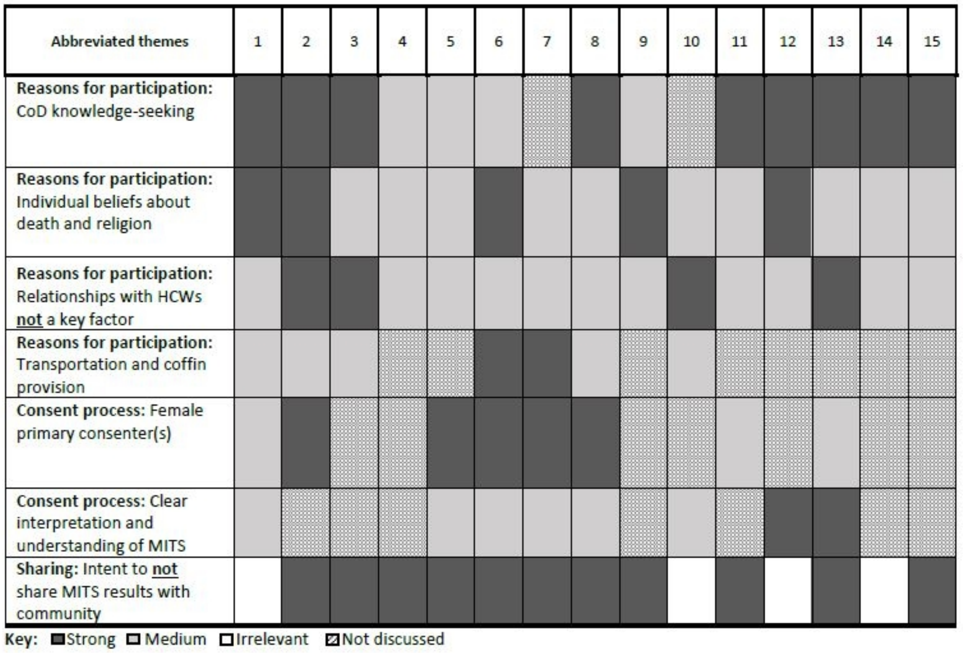

Figure 2. Strength of themes by family.

207x142mm (96 x 96 DPI)

Supplemental Table 1. Interview summaries.

Family #	Summary	Primary caregiver(s)	Caregiver(s) religion	Understood diagnosis available	Primary consenter(s)	Interview participant(s)
**1**	Family first went to multiple health centers, where they reported being poorly treated by HCWs. They enrolled in CHAIN in hopes their engagement in the study would improve the child's health. Family very pleased with care by CHAIN team generally. MITS participation was motivated by wanting to better understand child's CoD but did not understand MITS consent procedures well.	Mother and father	Pentecostal	Yes	Mother and father	Mother and father
**2**	Child's mother reported not getting along with the father. She had previously enrolled in malaria research and enrolled the child in CHAIN. Mother was pleased with CHAIN care but displeased with HCWs at night. She was motivated to know her child's CoD, especially if hereditary.	Mother	Pentecostal	No	Mother (grandmother present for support only)	Mother
**3**	Child's mother was displeased with care provided when child's condition was deteriorating. She consented independently to CHAIN but did not participate in the MITS consent process, deferring to tradition. Child's father and grandfather wanted to know CoD, thinking clinical diagnosis might not be the "real" CoD.	Mother	Protestant	Yes	Father and maternal uncle	Mother
**4**	Child was receiving care at a health center for a month before admission to QECH. Child was enrolled in CHAIN. Family was interested in learning CoD and did not want to take the time to solicit input from other family members during consent process.	Mother and father	Pentecostal	No	Father	Mother and father
**5**	Family did not want to seek care at local health center because they believed care quality there to be poor. Child was enrolled in CHAIN. Child's father had trouble visiting during admission because they lacked funds for transport. He was not involved in consent process. Child's maternal aunt was involved in the MITS consent and initially expressed concerns about organ harvesting. The mother wanted to know CoD.	Mother and father	Pentecostal	No	Mother, maternal aunt and cousin, and cousin's husband	Mother

For peer review only

**6**	Child's father left mother after family tested HIV positive and accused mother of sex work. He visited during child's admission at QECH but was not involved in the consent process. Child was enrolled in a nutrition substudy of CHAIN and mother felt supported by HCWs at QECH. Child's mother was interested in better understanding child's CoD but was primarily motivated by transport and coffin provision, as told to her by staff on the wards before the consent process began.	Mother	Catholic	Yes	Mother	Mother
**7**	Child was enrolled in a nutrition substudy of CHAIN. Family was influenced by transport provision in their decision to consent. Mother consented alone before the child's uncle arrived and he confirmed to proceed.	Mother	Catholic	No	Mother and grandmother	Mother, grandmother, and uncle
**8**	Child's mother was young (last year of secondary school) and initially hid her pregnancy. Child's father recently had a stroke and was receiving treatment. Family was very upset with care, especially lack of testing they were told was going to be done. Family's motivation to participate in MITS was to provide the CoD results to the father's family if they asked, but only if they asked. All three participants were from different faith groups (Islam, Protestant, and Catholic) but raised as Muslim.	Mother	Protestant	Yes	Mother, grandmother, and maternal aunt	Mother, grandmother, and maternal aunt
**9**	Child had sustained severe burns that were being managed on an outpatient basis prior to the last hospital admission. The father's family's church prohibits hospital treatment. However, family elects to seek treatment when needed and accepts church reprimands. While the mother was not involved in MITS consent process (she had returned home), she reported she would have consented as well.	Mother and father	Apostolic	Yes	Maternal aunt and her husband, and maternal uncle	Mother and father
**10**	Child's father visited QECH during the hospital stay. Both parents were dissatisfied with the way they were treated at hospital, and they were particularly upset the mother was never asked about child's condition. Father initially consented by phone. He did not seem to have a clear understanding of the MITS procedure. Consent motivated by feeling that caregivers should do as HCWs ask.	Mother and father	Catholic	No	Father	Mother and father

For peer review only

**11**	Family seemed satisfied with communication by HCWs, although limited. Family was keen to know more about CoD because they did not think the child would die as a consequence of pneumonia diagnosis. Child was buried the day after death and family thought they would have received pressure to not consent by the sub-chief if he would have been involved in decision-making.	Mother	Data unavailable	Yes	Father by phone, maternal grandmother, and great aunt	Mother and paternal grandmother
**12 (both twins enrolled in MiM)**	Child's mother was struggling with mental health condition and limited resources during twins' life. She had quit taking antiretroviral medications for HIV infection prior to their deaths at the recommendation of a local "prophet". Twins' father left their mother before their birth. The mother died shortly after twins' deaths and the uncle was interviewed. They had consented to MITS as a family, hoping it would alleviate blame for the twins' mother.	Mother	Apostolic	Data unavailable	Mother, maternal uncle, and maternal grandmother	Uncle
**13**	Child's mother was not satisfied with care received at a nearby public health center and at a private clinic, so she sought care at QECH without a referral. Family was satisfied with the care at QECH. Family consented to MITS to gain CoD information to protect surviving twin.	Mother and father	Pentecostal	No	Mother, father, paternal aunt, and female friend	Mother and father
**14**	Child's mother left child with father at 5 months after he married a second wife. Biological mother stayed with child at QECH, but grandparents (primary caregivers) did not seek mother's input during consent process because she was no longer the guardian. Health treatment was sought at a health center for child's condition prior to admission at QECH. Child was enrolled in a nutrition study. Family consented because they were interested in learning CoD.	Paternal grandparents	Islam	No	Paternal grandparents, (paternal aunt also present)	Paternal grandparents and stepmother
**15**	Child's father visited occasionally during QECH admission. He noted receiving no information from HCWs during visits. Family consented because they were interested in learning CoD and felt it would be helpful to their community, but they had no intention to share the results due to fears of rumors and misconceptions. They were relieved to receive transportation.	Mother and father	Protestant	Data unavailable	Uncle and father	Uncle and father

Abbreviations: CoD, cause of death; HCWs, healthcare workers; QECH, Queen Elizabeth Central Hospital

For peer review only

For peer review only

Appendix A: Discussion Guide

Number	Topic
T01.	**Introduction**<br>• Offering condolences to the family on the loss<br>• Thanking them for granting Consent to MITS as well as for accepting to be interviewed<br>• Briefing them of their rights (and support available) through the course of the interview<br>• Highlighting the essence of the interview with specific comments on the confidentiality of their responses
T02.	**Socio-demographic details**<br>• Verifying details provided at CHAIN recruitment<br>• Asking on additional information
T03.	**Exit Survey**<br>• Administering exit survey
T04.	**History of illness**<br>• Perceived origins/cause of illness<br>- Traditional causes/Medical causes<br>• Specific stages of illness (e.g. stage that made them decide to go to hospital, inform extended family, who decided on action to be taken etc)<br>• Admission into hospital<br>• Recruitment into the CHAIN study<br>- Consenting for tests while in hospital (including decision making around consent)<br>- Relationship with the main CHAIN team<br>• Previous experience of hospital care<br>• Social support in course of illness
T05.	**Hospital care post death**<br>• Explanation of CoD from the hospital<br>• Understanding of explained CoD<br>- Sufficiency of explained CoD<br>- Alignment with their initial thoughts on CoD<br>• Explained CoD ability to address concerns
T06.	**Approach for MITS**<br>• Consent for MITS<br>• Information given for MITS<br>• Reasons for Consenting to MITS

For peer review only - http://bmjopen.bmj.com/site/about/guidelines.xhtml

	• Decision making
T07.	**MITS and burial practices**
	• Religious and traditional customs observed in child death and burial<br>- Usual burial rites for child (age specific in relation to tradition/religion)<br>• MITS implication on observing customs
T08.	**Sharing of MITS information**
	• People told about the procedure and reasons for telling them<br>• Reactions of the people told, things heard in hospital or community<br>• Family and HH response to the reactions<br>-
T09.	**Knowledge production**
	• Knowledge gained after participating in MITS<br>• Impact of MITS in relating to hospital/HCWs<br>• Impact of MITS in future home care and care-seeking for illnesses<br>• Overall benefits from the procedure/experience
T10.	**Concluding remarks**
	• Regrets<br>• Recommendations for MITS consent approach<br>• Recommendations on releasing results of MITS to families<br>• Likelihood of recommending MITS<br>• General comments

For peer review only

COREQ (COnsolidated criteria for REporting Qualitative research) Checklist

A checklist of items that should be included in reports of qualitative research. You must report the page number in your manuscript where you consider each of the items listed in this checklist. If you have not included this information, either revise your manuscript accordingly before submitting or note N/A.

Topic	Item No.	Guide Questions/Description	Reported on Page No.
**Domain 1: Research team and reflexivity**			
Personal characteristics			
Interviewer/facilitator	1	Which author/s conducted the interview or focus group?	
Credentials	2	What were the researcher's credentials? E.g. PhD, MD	
Occupation	3	What was their occupation at the time of the study?	
Gender	4	Was the researcher male or female?	
Experience and training	5	What experience or training did the researcher have?	
Relationship with participants			
Relationship established	6	Was a relationship established prior to study commencement?	
Participant knowledge of the interviewer	7	What did the participants know about the researcher? e.g. personal goals, reasons for doing the research	
Interviewer characteristics	8	What characteristics were reported about the inter viewer/facilitator? e.g. Bias, assumptions, reasons and interests in the research topic	
**Domain 2: Study design**			
Theoretical framework			
Methodological orientation and Theory	9	What methodological orientation was stated to underpin the study? e.g. grounded theory, discourse analysis, ethnography, phenomenology, content analysis	
Participant selection			
Sampling	10	How were participants selected? e.g. purposive, convenience, consecutive, snowball	
Method of approach	11	How were participants approached? e.g. face-to-face, telephone, mail, email	
Sample size	12	How many participants were in the study?	
Non-participation	13	How many people refused to participate or dropped out? Reasons?	
Setting			
Setting of data collection	14	Where was the data collected? e.g. home, clinic, workplace	
Presence of non-participants	15	Was anyone else present besides the participants and researchers?	
Description of sample	16	What are the important characteristics of the sample? e.g. demographic data, date	
Data collection			
Interview guide	17	Were questions, prompts, guides provided by the authors? Was it pilot tested?	
Repeat interviews	18	Were repeat inter views carried out? If yes, how many?	
Audio/visual recording	19	Did the research use audio or visual recording to collect the data?	
Field notes	20	Were field notes made during and/or after the inter view or focus group?	
Duration	21	What was the duration of the inter views or focus group?	
Data saturation	22	Was data saturation discussed?	
Transcripts returned	23	Were transcripts returned to participants for comment and/or	

For peer review only

Topic	Item No.	Guide Questions/Description	Reported on Page No.
		correction?	
**Domain 3: analysis and findings**			
Data analysis			
Number of data coders	24	How many data coders coded the data?	
Description of the coding tree	25	Did authors provide a description of the coding tree?	
Derivation of themes	26	Were themes identified in advance or derived from the data?	
Software	27	What software, if applicable, was used to manage the data?	
Participant checking	28	Did participants provide feedback on the findings?	
Reporting			
Quotations presented	29	Were participant quotations presented to illustrate the themes/findings? Was each quotation identified? e.g. participant number	
Data and findings consistent	30	Was there consistency between the data presented and the findings?	
Clarity of major themes	31	Were major themes clearly presented in the findings?	
Clarity of minor themes	32	Is there a description of diverse cases or discussion of minor themes?	

Developed from: Tong A, Sainsbury P, Craig J. Consolidated criteria for reporting qualitative research (COREQ): a 32-item checklist for interviews and focus groups. *International Journal for Quality in Health Care*. 2007. Volume 19, Number 6: pp. 349 – 357

Once you have completed this checklist, please save a copy and upload it as part of your submission. DO NOT include this checklist as part of the main manuscript document. It must be uploaded as a separate file.

For peer review only

BMJ Open

Primary motivations for and experiences with pediatric minimally invasive tissue sampling (MITS) participation in Malawi: A qualitative study

Journal:	*BMJ Open*
Manuscript ID	bmjopen-2021-060061.R1
Article Type:	Original research
Date Submitted by the Author:	26-Apr-2022
Complete List of Authors:	Lawrence, Sarah; University of Washington, Pediatrics<br>Namusanya, Dave; Abertay University, Behaviour and Health Research Group; Malawi-Liverpool-Wellcome Trust Clinical Research Programme, Behavior and Health Research Group<br>Mohamed, Sumaya; University of Washington, Global Health<br>Hamuza, Andrew; Malawi-Liverpool-Wellcome Trust Clinical Research Programme, Behaviour and Health Research Group<br>Huwa, Cornelius; University of Malawi College of Medicine, Paediatrics<br>Chasweka, Dennis; University of Malawi College of Medicine, Paediatrics; The Childhood Acute Illness & Nutrition Network<br>Kelley, M; University of Oxford, Health Systems Collaborative, Nuffield Department of Medicine; The Childhood Acute Illness & Nutrition Network<br>Molyneux, Sassy ; University of Oxford, Health Systems Collaborative, Nuffield Department of Medicine ; The Childhood Acute Illness & Nutrition Network<br>Voskuijl, Wieger ; Amsterdam University Medical Centres, Pediatrics; The Childhood Acute Illness & Nutrition Network<br>Denno, Donna; University of Washington, Department of Pediatrics; The Childhood Acute Illness & Nutrition Network<br>Desmond, Nicola; Liverpool School of Tropical Medicine, International Public Health; Malawi-Liverpool-Wellcome Trust Clinical Research Programme, Behaviour and Health Research Group
**Primary Subject Heading**:	Global health
Secondary Subject Heading:	Qualitative research, Paediatrics, Public health
Keywords:	PAEDIATRICS, TROPICAL MEDICINE, PUBLIC HEALTH, Anthropology < TROPICAL MEDICINE

SCHOLARONE™ Manuscripts

I, the Submitting Author has the right to grant and does grant on behalf of all authors of the Work (as defined in the below author licence), an exclusive licence and/or a non-exclusive licence for contributions from authors who are: i) UK Crown employees; ii) where BMJ has agreed a CC-BY licence shall apply, and/or iii) in accordance with the terms applicable for US Federal Government officers or employees acting as part of their official duties; on a worldwide, perpetual, irrevocable, royalty-free basis to BMJ Publishing Group Ltd ("BMJ") its licensees and where the relevant Journal is co-owned by BMJ to the co-owners of the Journal, to publish the Work in this journal and any other BMJ products and to exploit all rights, as set out in our licence.

The Submitting Author accepts and understands that any supply made under these terms is made by BMJ to the Submitting Author unless you are acting as an employee on behalf of your employer or a postgraduate student of an affiliated institution which is paying any applicable article publishing charge ("APC") for Open Access articles. Where the Submitting Author wishes to make the Work available on an Open Access basis (and intends to pay the relevant APC), the terms of reuse of such Open Access shall be governed by a Creative Commons licence – details of these licences and which Creative Commons licence will apply to this Work are set out in our licence referred to above.

Other than as permitted in any relevant BMJ Author's Self Archiving Policies, I confirm this Work has not been accepted for publication elsewhere, is not being considered for publication elsewhere and does not duplicate material already published. I confirm all authors consent to publication of this Work and authorise the granting of this licence.

For peer review only

Primary motivations for and experiences with pediatric minimally invasive tissue sampling (MITS) participation in Malawi: A qualitative study

Sarah Lawrence[1†], Dave Namusanya[2,3†], Sumaya B. Mohamed[4], Andrew Hamuza[3], Cornelius Huwa[5,6], Dennis Chasweka[5,6], Maureen Kelley[6,7], Sassy Molyneux[6,7], Wieger Voskuijl[5,6,8,9], Donna M. Denno[1,4,6], Nicola Desmond*[3,10]

† shared first authorship

[1] Department of Pediatrics, University of Washington, Seattle, Washington, United States of America

[2] Behaviour and Health Research Group, Abertay University, Dundee, United Kingdom

[3] Behaviour and Health Research Group, Malawi-Liverpool-Wellcome Trust, Blantyre, Malawi

[4] Department of Global Health, University of Washington, Seattle, Washington, United States of America

[5] Department of Paediatrics and Child Health, Kamuzu University of Health Sciences (formerly College of Medicine), Blantyre, Malawi

[6] The Childhood Acute Illness & Nutrition Network, Nairobi, Kenya

[7] Health Systems Collaborative, Nuffield Department of Medicine, University of Oxford, United Kingdom and KEMRI-Wellcome Trust Research Programme, Kilifi, Kenya

[8] Amsterdam Institute for Global Health and Development, Department of Global Health, Amsterdam University Medical Centres, Amsterdam, The Netherlands

[9] Amsterdam Centre for Global Child Health, Emma Children's hospital, Amsterdam University Medical Centres, Amsterdam, The Netherlands

[10] Department of International Public Health, Liverpool School of Tropical Medicine, Pembroke Place, Liverpool, United Kingdom

* Corresponding author

Email: nicola.desmond@lstmed.ac.uk (NK)

Word Count: 4901

For peer review only
For peer review only - http://bmjopen.bmj.com/site/about/guidelines.xhtml

ABSTRACT

Objective: To understand family member consent decision-making influences and experiences in Malawi in order to inform future MITS studies.

Design: Qualitative study.

Setting: Queen Elizabeth Central Hospital (QECH) in Blantyre, Malawi, which serves as the central referral hospital for southern Malawi and where MITS participants were recruited from.

Participants: Families of pediatric MITS participants.

Methods: We conducted in-depth interviews with 16 families six weeks after the death of pediatric MITS participants. Data were analyzed using a combination of thematic content and theoretical framework approaches to explain the findings.

Results: Improved CoD ascertainment was the principal motivator for participation to protect remaining or future children. Community burial norms, religious doctrine, and relationships with healthcare workers were not reported influencers among family members who consented to the procedure. Primary consenters varied, with single mothers more likely to consent independently or with only female family members present. Clear understanding of MITS procedures appeared limited six weeks post-procedure, but research was described as voluntary and pre-consent information satisfactory for decision-making. Most families intended to share about MITS only to those involved in the consent process, for fear of rumors or judgment by extended family members and the wider community.

Conclusion: Among those who consented to MITS, decision-making was informed by individual and household experiences and beliefs, but not by religious affiliation or experiences with HCWs. While

For peer review only

understanding of the MITS procedure was limited at the time of interview, families found informed consent information sufficient for decision-making. Future MITS studies should continue to explore information presentation best practices to facilitate informed consent during the immediate grieving period.

ARTICLE SUMMARY

Strengths and limitations of this study

- This study conducted in-depth interviews with families following MITS participation to improve understanding of MITS decision-making factors, experiences with the consent process, and plans for MITS results utilization.
- The MiM study was pediatric and hospital-based, therefore findings may not be generalizable to adult or community-based MITS studies or to settings outside of southern Malawi.
- We lack in-depth data from families who declined to consent to MITS, hence our detailed findings only reflect the views shared by family members who consented to the study.

INTRODUCTION

Improved cause of death (CoD) determination is essential to tailor intervention development and implementation to reduce preventable child deaths. Complete diagnostic autopsy (CDA) is the gold standard for determining CoD but is often challenging or unfeasible due to resource constraints, limited acceptability linked to cultural and social norms, and concerns about body disfigurement. In response to

For peer review only - http://bmjopen.bmj.com/site/about/guidelines.xhtml

these barriers, minimally invasive tissue sampling (MITS) has been validated and is increasingly being utilized as an alternative to CDA to establish CoD in various settings, including among child deaths.[1, 2] MITS uses small needles to sample organs and body fluids for histopathological and microbiologic investigations to inform CoD determinations.[3]

Due to the sensitivity of the procedure, the immediate personal tragedy faced by parents/caregivers from whom consent is sought, and the need to evaluate context-specific acceptability, pre-implementation formative acceptability research has been widely implemented.[4-11] MITS has generally been found to be more acceptable than CDA across diverse settings, reducing some of the cultural, social, and structural restrictions that constrain CDA implementation.[4-6, 12, 13] In particular, body disfigurement—a commonly cited reason for declining CDA – is mitigated.[4-6, 13] Formative research to understand *hypothetical* pediatric MITS acceptability in Malawi, found that acceptability would likely be strongly influenced by social relationships between parents, extended family, hospital workers, and research staff, but that religious affiliation would be less influential than individual and household beliefs and practices.[14] Participants believed MITS participation would be facilitated by desire for improved CoD information but hindered by fears of organ harvesting and disruption to transportation and burial plans, corroborating evidence from similar studies in other settings.[10, 15] Body disfigurement was still raised as a concern in the Malawi formative assessment.[14]

While evidence on MITS acceptability derived from pre-implementation assessments has increased in recent years, post-participation assessments have been limited.[8] With increasing MITS utilization globally, it is important to assess how families perceive the procedures after participation to optimize consent and support processes for grieving families.

For peer review only - http://bmjopen.bmj.com/site/about/guidelines.xhtml

METHODS

Study setting

The MITS in Malawi (MiM) study was conducted at Queen Elizabeth Central Hospital (QECH) which serves as the central referral hospital for southern Malawi. Services are largely provided free of charge. Eligibility included children (7 days to 4 years) who died during hospitalization with acute illness and/or malnutrition and with a primary caregiver residing within a 50-kilometer radius of QECH. This catchment area includes ethnically diverse, predominantly Christian, matrilocal urban and rural communities.

MiM started as a sub-study of the Childhood Acute Illness & Nutrition (CHAIN) Network, which aims to identify mortality risk factors among children hospitalized with acute illness or undernutrition across nine sites in six countries,[16] but was expanded to patients enrolled in two other undernutrition studies conducted by the same research group and to the general pediatric wards. Caregivers approached for enrollment were offered transportation and a coffin after information about the study was provided, irrespective of participation decision.

Study design and sampling

In-depth interviews were conducted with family members who consented to the MITS procedure to understand experiences with the consent process, decision-making factors, and their plans for using and sharing of MITS results. During the consent for MITS, written consent was obtained from the family for interviews six weeks later at QECH or at their home, whichever they preferred. Transport for participants to and from the interview was provided by the study. Interview participants were

For peer review only - http://bmjopen.bmj.com/site/about/guidelines.xhtml

determined by the family and family members interviewed together as is natural in the cultural context (see Table 1). Those who did not consent to MITS were asked their reasons for withholding consent.

A discussion guide (Appendix A) was developed in English, based on formative qualitative research exploring MITS acceptability in Malawi.[14] It was translated to Chichewa (the local language). Sociodemographic data was available from linked case report forms for participants co-enrolled in other studies and additional sociodemographic data was collected during the interview. Interviews focused on experiences within the hospital prior to death, interactions with the MITS study team, consent procedures, motivations for participation, and intended use of MITS results.

Data collection and analysis

Interviews were conducted between September 2018 and December 2019. All participants preferred to receive MITS results and be interviewed at QECH. Participants first met with the study team to receive CoD results. They then provided verbal consent to be interviewed. Interviews were conducted by a male, masters-level social scientist (DN, shadowed by AH) who had not previously met the participants and was introduced by the clinical MITS study team member who provided CoD results and answered any questions about the findings. Interviews were conducted in Chichewa largely using a narrative approach led by participants while addressing discussion guide topics. Interviews were recorded, transcribed, and translated verbatim. Post-interview reflections (by DN) were noted to capture key topics and initial thoughts.

Transcripts were analyzed using a codebook that was developed iteratively through reading post-interview reflection reports and transcripts, code development and refinement, preliminary code application to transcripts, discussion of code application, and revision to the codebook. Transcripts were

For peer review only - http://bmjopen.bmj.com/site/about/guidelines.xhtml

imported into NVivo 11 Plus (QSR International Pty Ltd). Each transcript was coded independently by one team member (DN, SL, SBM). Transcripts were exchanged and coded transcripts were reviewed by a second coder. Coding disagreements were resolved through group discussion.

Interviews were analyzed using thematic content and framework analysis to produce a description of key themes across all interviews.[17, 18] Coded data were grouped into themes and then explored by socio-demographic features, such as primary caregiver, past experiences in research, and religion to identify if key characteristics influenced MITS decisions, participation experiences, and reflections following CoD results provision. Individual case study descriptions were also produced to explore situated experiences in-depth, drawing on a phenomenological approach that recognizes the unique experiences of individuals, especially where there is little prior knowledge available about the topic.[19]

Ethics approvals

The Malawi National Health Sciences Research Committee (Protocol #17/09/1913) and the Oxford Tropical Research Ethics Committee (Reference 34-16) approved the study protocols. The University of Washington Institutional Review Board (STUDY00003689) exempted the study from review. Written informed consent was provided by all participants.

Patient and public involvement

Patients or the public were not involved in the design, or conduct, or reporting, or dissemination plans of our research.

RESULTS

For peer review only - http://bmjopen.bmj.com/site/about/guidelines.xhtml

For peer review only

Of the 58 families approached for participation in MiM, 29 (50%) consented (Figure 1). Families most often cited lack of benefits as the primary refusal reason – if they were already satisfied with the CoD information provided – followed by not wanted to delay burial, and religious concerns including beliefs that death is God's will and therefore needs no further investigation or that postmortem procedures are explicitly prohibited by their specific place of worship. Two families were disappointed in the care their child received during hospitalization and felt more should have been done while the child was alive, not research after death. Two other families were unable to reach the family member with the authority to consent (as defined by the family – father and uncle for one case and biological mother for another) and another family felt their child was too young (2 weeks old) to have postmortem procedures conducted.

Figure 1. Consent to MITS and interviews.

Of the 29 families who consented to the MITS procedures, 16 participated in interviews. Nine families were not approached for interview due to social scientist unavailability (cases 21-29) and three could not be reached for interview and results provision. 16 of 16 families approached for interviews consented. One interview was omitted due to poor audio quality. Interviews included one to three family members as preferred by participants, most often the mother and father together if both were available (Table 1). Interviews lasted between 31 and 91 minutes.

Table 1. Characteristics of participants (n=15).

Characteristic	Median (IQR) or n (%)
Relationship to MITS participant	
Mother & father	5 (33.3)
Mother	4 (26.7)
Mother, grandmother, & aunt/uncle	3 (20.0)
Father & uncle	1 (6.7)
Uncle	1 (6.7)

For peer review only - http://bmjopen.bmj.com/site/about/guidelines.xhtml

Stepmother & grandparents	1 (6.7)
Prior child death in family	
Yes	1 (12.5)
No	4 (26.7)
N/A - first child	4 (26.7)
Data unavailable*	6 (40.0)
Primary caregiver's religion	
Pentecostal	5 (33.3)
Catholic	3 (20.0)
Protestant	3 (20.0)
Apostolic	2 (13.3)
Islam	1 (6.7)
Data unavailable*	1 (6.7)
Prior research participation	
CHAIN or other nutrition study	8 (53.3)
Other research prior to admission	1 (6.7)
Data unavailable*	6 (40.0)
Reported informed of child's diagnosis during life	
Yes	6 (40.0)
No	7 (46.7)
Data unavailable*	2 (13.3)
**Characteristics of MITS participant**	
Female	4 (26.7)
Age (months)	7.6 (3.0, 16.3)
Living twin	2 (12.5)
Primary caregiver	
Mother	6 (40.0)
Mother & father	8 (53.3)
Grandparents	1 (6.7)
Number of living biological siblings	
3+	5 (33.3)
2	2 (13.3)
1	1 (6.7)
0	6 (40.0)
Data unavailable*	1 (6.7)
Inpatient stay duration (days)	4.0 (2.0, 8.0)

*not discussed in interview with family

Definitions: MITS, minimally invasive tissue sampling; N/A, not applicable

Participant characteristics of each are summarized in Supplemental Table 1, including reasons for participating in MITS and consent dynamics. Socio-demographic characteristics, such as primary caregiver and religion, were unrelated to factors described as influencing decision-making, amongst those who participated.

For peer review only - http://bmjopen.bmj.com/site/about/guidelines.xhtml

For peer review only

For those who consented, MITS participation was largely reported to be influenced by desire for improved CoD knowledge, individual beliefs about burial traditions and religious practices, and in some instances, transportation and coffin provision. Participants reported that interactions with health care workers (HCWs) or researchers, either positive or negative, did not influence their decision-making. Primary consenters were identified by families and varied depending on each particular situation. Clear understanding of the MITS procedure was often limited at the time of interview. Most participants did not intend to share MITS results outside of those involved in the consent process. The strength of themes varied across interviews (Figure 2).

Figure 2. Strength of themes by family.

Reasons for MITS research participation

Knowledge-seeking

Desire for improved CoD information was the primary driver of MITS participation. Caregivers saw MITS as an opportunity to learn about the cause of illness and subsequent death, especially when a clear, well-understood diagnosis was not reported as provided during the child's life. Most families provided with diagnoses during the child's life believed the information was insufficient, due to limited information provided by HCWs or their own limited understanding. They often believed this prior information was not representative of the "true" CoD.

For peer review only - http://bmjopen.bmj.com/site/about/guidelines.xhtml

…if we refused [MITS], we would not have known the cause of the death because we were just thinking that it's just mere diarrhea. We didn't really know the disease, so I wanted to know the cause of death. (Mother, Family 2)

For most families, CoD information-seeking was driven by a desire to protect remaining or future children through identification of hereditary illnesses or insights that might guide child-rearing practices. Two families reported motivation to protect the life of the living twin, both of whom also had two or more other living siblings.

We were grappling with questions as parents and we were wondering that with this sort of death, then how best can we take care of the surviving twin child. So we said, let us consent and then they can find out the actual cause of death and if it will help others then also it will help us as parents. (Father, Family 1)

Five participants also identified MITS as beneficial to the wider community through its potential to inform the development of future treatments so others will not have to suffer the loss of a child.

We agreed to do postmortem so that the findings could help someone in future. We don't want what happened to us to happen to other people. (Father, Family 13)

Individual beliefs about burial and religion

Participants shared that their decision to participate was strongly influenced by individual beliefs about death and religion rather than by broader community burial norms or religious doctrine.

Burial

For peer review only - http://bmjopen.bmj.com/site/about/guidelines.xhtml

Participants reported they were not concerned with potential burial delays or morticians cleaning the body, instead of following traditional burial practices whereby the family cleans the body prior to burial. Practices regarding burial traditions were seen as guidelines, more than rules.

> *We are used to washing the body before burial so when we arrived at the village and the people asked about washing the body, we told them that the body had already been washed and they said that it is not a problem.* (Mother, Family 1)

Some families whose child died in the late afternoon elected to wait for the MITS procedure to be conducted the next morning, even though burials are usually conducted on the same day. These participants were particularly motivated to learn CoD information that they hoped MITS would provide and felt that travel by night would be logistically difficult. They did not view waiting for MITS as a significant breach of tradition. Although, ensuring prompt burial was the primary reason stated for refusing participation in MITS for some families (Fig 1).

Religion

Interviewees viewed MITS participation benefits as outweighing community expectations and norms defined by particular religious affiliation. For example, one family with membership in an Apostolic church whose members usually oppose biomedical health care chose to both seek inpatient and outpatient medical treatment when their child was severely burned, but to also participate in MITS to learn more about the child's death.

For peer review only - http://bmjopen.bmj.com/site/about/guidelines.xhtml

They [church leaders] say that do not go to the hospital but it's up to you the patient to decide based on how you are feeling…you just discipline yourself [after going to the hospital]. (Mother, Family 10)

Religious affiliations were not known for all of the five families who declined MITS and cited religious or cultural reasons as their primary concern, although one, for example, noted their Presbyterian church does not allow procedures on the deceased. Another noted they do not believe further investigation is needed after death because death is the will of God.

Relationships with HCWs

Communication with HCWs and research staff, regardless of whether deemed to be satisfactory, did not emerge as a primary factor in consent decision-making for participants. They had variable experiences with HCWs during the QECH inpatient stay and at prior health facilities. Most participants noted some rudeness, limited communication, or lack of follow through by HCWs at some point in their child's care but were ultimately motivated by improved CoD understanding. Participants whose child was enrolled in research studies during life mentioned that researchers exhibited more compassion and attentiveness toward them than clinical staff on the wards.

When I came to the hospital and met with doctors from CHAIN, I could see that they would talk to me with respect; yet when I met with the others, the doctors from the wards, they just spoke to me rudely. They just spoke as if they are tired with their job. (Father, Family 1)

Participants acknowledged they did not usually ask questions of HCWs on the wards, rather they merely observed, because HCWs have authority over information regarding their child's treatment.

For peer review only - http://bmjopen.bmj.com/site/about/guidelines.xhtml

They did not tell us [about the child's treatment], we were just watching being that they are the doctors. (Grandmother, Family 8)

A few of the participants shared they were counseled about their child's deteriorating health and imminent death, although others voiced concerns that HCWs had not responded to their child's deterioration.

While families had mixed experiences with HCWs prior to the child's death, none identified this as a primary factor in deciding to consent to MITS. However, two families declined MITS due to frustration with the care provided during the child's life.

Nobody came, they were just busy playing with their phones, but they saw that I was stressed up with the child, they were just looking at him, maybe they knew that the child was dying…. (Mother, Family 10)

Transportation and coffin provision

HCWs involved in care during the inpatient stay introduced the family to MiM research team. A few families noted they were advised by HCWs that the MiM study presented an opportunity for transportation and a coffin, and this strongly influenced their consent decision-making.

What made me to accept was that I wanted to know the cause of the death because he was having diarrhea but also, I wanted to be helped with transport to get home. (Mother, Family 6)

For peer review only - http://bmjopen.bmj.com/site/about/guidelines.xhtml

When the research team became aware of this practice by some HCWs partway through the study, a hospital staff re-training was implemented to reinforce that transport and coffins would be provided regardless of consent and offered to the families by the research team after approach for consent was complete. The finding of this influencer was no longer observed in interviews conducted subsequent to the re-training (post Family 11).

MITS consent dynamics

Primary consenters

Household norms and familial support in the hospital informed who was involved in the MITS consent process and who retained the ultimate authority to consent or not. Most primary caregivers consulted other family members during the decision-making, often including the child's grandparents, uncles, and aunts – especially when they lived nearby or were involved in the child's care during life. When multiple family members were involved, the primary decision-maker for MITS often was the male head of household or the child's maternal uncle.

> *I could not give consent on this on my own…we [researcher and grandmother] left together with my aunt and sat beside the mortuary, we [grandparents] linked up. After linking up, it was found that the dad [child's grandfather] agreed and said he had given consent on this.* (Grandmother, Family 14)

For peer review only - http://bmjopen.bmj.com/site/about/guidelines.xhtml

For peer review only

Some parents did not consult with extended family members, as they believed they held the sole responsibility of their child, including after death. Place of residence (rural vs. peri-urban) did not influence the primary consenter choice within the family.

> *We did not go to consult because we are the parents, the responsibility towards the child rests with us; that is why we just accepted that they should go ahead because we also wanted to know what the problem was.* (Father, Family 1)

Mothers were more likely to consent alone if the father was uninvolved in the child's care generally or if there were marital difficulties.

> *I did everything on my own. There was nobody to discuss with. I was like a father and a mother, so I was supposed to make decisions on everything.* (Mother, Family 2)

Single mothers were also more likely to only engage women in the consent process, often their mothers and sisters who were supporting them during the child's hospital stay or first to arrive. One woman who had a strained relationship with the child's father consented with the child's maternal grandmother present, who had been with her during the child's hospitalization, and then later informed the father of the decision, but she did not feel he needed to be involved in the process.

Interpretation and understanding

Interviewees were often unable to describe the MITS procedure. The only component one participant recalled (erroneously) was the removal of the brain, noting that this was necessary to determine the

For peer review only

CoD. She reported satisfaction with the information provided during the consent process but could not recall further details. Another participant described the procedure as an “x-ray”. Two participants highlighted MITS would test more than one area of the body–one noted three to four areas would be tested, while another that “each and every part of the body” would be tested. Only two participants explicitly noted that MITS involves needle sampling and no incisions.

> *They explained to me saying: if possible, there is need to examine the bodies of the children, we will not tear the bodies in any way, but we just use some needles. So that, once we find out about something in the future, since we still have a generation, maybe this can be something to say: we should get prepared now, before something happens. Therefore, according to the way they explained, I felt they gave in some good points.* (Uncle, Family 12)

> *The researchers said their research is not about cutting the body anywhere. They said they will use an injection to pull out fluid from the nerves or from the spinal cord. They said they will get these samples from 3 or 4 areas. When they get these samples, they will tell us the results after one month. I told them, there is no problem. They said after taking the samples, we will ask you to go and confirm that we have not cut anywhere.* (Father, Family 13)

Across all the participants who described the consent procedures, they understood MITS aimed to provide additional information about the child’s CoD and their participation was not mandatory.

> *So the hospital people came saying that if the death happening like this [you can participate in MITS] but it’s not a compulsory thing. We take the body to the mortuary for MITS to find out the real cause of the death.* (Mother, Family 8)

For peer review only - http://bmjopen.bmj.com/site/about/guidelines.xhtml

Sharing MITS results

Participants reported being satisfied with the CoD results received from the MITS research team and were able to explain the findings and recommendations provided. While many participants who consented to MITS identified better understanding of their child's CoD as protective for their households and communities, very few of them planned to share information about their participation or results with extended family, neighbors, or other community members. Individuals involved in the consent process were frequently the only individuals with whom parents and caregivers planned to share the results. Participants shared this decision was influenced by fear of what people might say about the procedures and the family themselves.

> *Somebody who may not be close, once you tell them, instead of them to understand, they begin asking you questions* (Grandmother, Family 14). *And they can even tell you that you could be the one responsible for my child's condition, nowadays.* (Stepmother, Family 14)

One participant noted that by telling even one relative, the family risked having their decision shared across the community when that relative tells others. Due to concerns that this potential for gossip would cause issues for the family, he decided not to share about MITS.

A couple of participants shared they would not have told their family members about MITS had they not already been present at the hospital. Some participants reported telling extended family they had enrolled in a study that was providing transport but did not elaborate further on research activities to avoid questions or judgement. Relatives and community members did not push for additional details

For peer review only - http://bmjopen.bmj.com/site/about/guidelines.xhtml

because transportation for deceased is often a challenge and this answer avoided what participants considered unwelcome questions.

Only four families informed uncles, traditionally regarded as heads of the extended family, that MITS had been conducted.

> *I will tell members of my extended family since they are my nephews and nieces…so they should know what caused the child's death.* (Father, Family 10)

The four families also reported they intend to share information learned about the CoD with these uncles. Of the four, one participant was primarily motivated to share results to prevent blame within the family.

> *People should be able to understand to say: it happened this way, the main issue was that this person died of this problem, so that there should not be finger pointing towards one another.* (Uncle, Family 12)

DISCUSSION

Our findings demonstrate that individual experiences and familial relationships are central to MITS consent and participation processes. Socio-demographic characteristics did not clearly influence decision-making themes, consent processes, or intention to share results. One notable exception was that single mothers were more likely to consent to MITS independently or with only female family

For peer review only - http://bmjopen.bmj.com/site/about/guidelines.xhtml

members present, possibly explained by the matrilocal setting.[20] In cases where the father was at least somewhat involved, they were engaged in the consent process and often had the principal authority to consent, corroborating hypothetical findings from formative MITS acceptability research in Mozambique, South Africa, Bangladesh, India andPakistan.[6, 8, 9, 21, 22] Interestingly, relationships with HCWs or research staff (neither positive nor negative) or dissatisfaction with the healthcare provided, did not seem to strongly influence decision-making among MiM participants, even though many did report negative interactions. This is in contrast to qualitative work pre-MiM study implementation from our group and other formative studies that suggested trust in the healthcare system and relationships with HCWs and research staff would be important drivers of consent.[14, 21] However, the data from our in-depth interviews are only among those who *consented to MITS* and reflect families' experiences six weeks after the death and MITS procedure.[4, 5, 14] Nevertheless, for these families, it was apparent that desire for improved CoD knowledge outweighed any negative experiences during the hospital stay and participants seemed to accept limited communication with HCWs as the norm. Participants who had received a clear diagnosis of the child's illness prior to death continued seeking additional information after death in a continuous search for meaning, reflecting similar qualitative findings in Tanzania where families continue negotiation in defining illness after death.[23]

While participants often identified MITS results as beneficial to surviving children and potentially protective to the broader community, they frequently did not intend to share their participation experiences or results outside of close family members involved in the consent process. Families were fearful of being asked unwanted questions, rumors about their family, and judgment about their participation. This reflects similar findings to formative MITS research in Pakistan that found women fear unexpected CoD results or that new, stigmatized diagnoses would result in blame toward them and the

For peer review only - http://bmjopen.bmj.com/site/about/guidelines.xhtml

possibility of abandonment by their husbands.[9] MITS researchers need to be acutely aware of inadvertent social harms that CoD results might bring to family members, particularly mothers. Similar to HIV research, MITS investigators should prioritize risk/benefit analyses of MITS participation to promote research participant welfare.[24, 25] As MITS becomes more widely used, experiences of families who have consented to MITS will potentially contribute to wider community acceptability. If families are unable to share their experiences due to fear of social harms, this could negatively influence MITS uptake and normalization of the procedure.

One concerning finding was that some participants were informed, prior to consent approach, by HCWs outside of the research team, of transportation and coffin provision through the MiM study, raising concerns of tacit coercion, albeit driven by in-hospital rumor rather than direct and purposeful communication from study staff. Non-study HCWs presented the study as an opportunity for families with limited resources to receive assistance–trying to help families in their time of need while going against the research protocol which outlined offering transportation and coffin after the approach for consent and regardless of the consent decision. Re-training of HCWs regarding the MiM consent protocol appears to successfully mitigate this practice. Research participation in resource limited settings is often influenced by perceived and actual benefits.[26] Transportation is a much-needed service in Malawi, especially after death because public transit will not transport a body, resulting in transport provision alone to be a sufficient explanation for research participation. This raises ethical concerns around undue inducement, while also highlighting the acute need for support and resources after losing a loved one.[27, 28] However, fear of undue inducement can lead to under-compensation or failure to offer essential support for families facing significant difficulties. As such, future MITS studies should carefully consider the benefits provided to all families (regardless of participation) to minimize undue inducement and also support families in need. Researchers in general should carefully monitor

For peer review only

For peer review only - http://bmjopen.bmj.com/site/about/guidelines.xhtml

how potential participants are introduced to the study team and whether benefits are presented in advance of the informed consent procedures by non-researchers, especially if compensation is only provided for those that consent.

Participants' limited ability to describe the MITS procedures six weeks later also raises questions about the ability to process information soon after the loss of a child and to revisit that information later. One study examining five other MITS sites in sub-Saharan Africa and South Asia reported emotional turmoil after loss of a loved one associated with limited understanding of the procedure during the consent process, requiring additional time for deliberation to consent.[29] While interviewees in this study reported understanding participation as voluntary, they focused more on the benefits and potential outcome of the procedures (improved CoD determination) rather than the procedure itself. Prior to study implementation, formative research was conducted through focus group discussions with HCWs, researchers, community leaders, and parents as well as discussions between researchers to establish how to best present the procedures – including the level of detail – to families.[14] However, interview findings demonstrated that understanding was still limited, but participants generally perceived their understanding of information about the MITS procedure during informed consent processes as satisfactory. Other research studies conducted with bereaved parents in Australia and Canada have found that the majority of individuals feel comfortable making informed consent decisions soon after the loss of a child.[30, 31]

This study has several limitations, including that we were only able to approach the first two-thirds of families for interview participation and the loss of interview data for one participant due to poor audio recording quality resulting in a small sample size. However, 100% of families approached did participate in post-procedure interviews and despite the small sample size data saturation was reached since no

For peer review only

For peer review only - http://bmjopen.bmj.com/site/about/guidelines.xhtml

new themes emerged during iterative reviews of the data collected. The MiM study was pediatric and hospital-based, hence findings may not be generalizable to adult or community-based MITS studies or to settings outside of southern Malawi. Most importantly, we lack in-depth data from families who declined to consent to MITS, hence our detailed findings only reflect the views shared by family members who consented to the study.

Despite these limitations, this study provides an improved understanding of MITS decision-making factors, experiences with the consent process, and plans for MITS results utilization by family members. The importance of improved CoD determination was highlighted as an important benefit of MITS that supersedes some potential barriers to participation, including negative interactions and poor communication with HCWs. This study highlights the importance of monitoring MITS and other studies for unintended undue inducement while also providing adequate support for grieving families with limited financial resources. The study also raises important questions around best practices for information provision in MITS studies to balance sufficient detail for informed consent during an emotionally turbulent period when such content may be less easily absorbed and/or remembered. As MITS studies become more prevalent, the ethical issues raised in this study should continue to be explored and delineated, including across different geographic and cultural contexts.

FUNDING STATEMENT

This study was funded by the Bill & Melinda Gates Foundation (https://www.gatesfoundation.org/) as a sub-study, led by DD and WV, of the CHAIN Network (grant number OPP1131320). Additional funding for MK and SM provided by a Wellcome Trust and MRC Newton Fund Collaborative Award

For peer review only

(https://mrc.ukri.org/news/browse/wellcome-trust-mrc-newton-fund-collaboration/)(grant number 200344). The funders had no role in study design, data collection and analysis, decision to publish, or preparation of the manuscript.

COMPETING INTERESTS

None declared.

DATA AVAILABILITY

The data that support the findings of this study are available on request from the corresponding author, ND. The data are not publicly available due to their containing information that could compromise the privacy of research participants.

ACKNOWLEDGEMENTS

We would like to thank participants for sharing their thoughts and experiences, particularly during a grief-marked period. We would also like to thank Hendrina Kaliati of the Malawi Liverpool Wellcome Trust Clinical Research Programme for transcription and translation of recorded data. We thank the Malawi CHAIN team and the CHAIN Network Principal Investigators, Prof. Judd L Walson and Prof. James A Berkley for their support.

For peer review only - http://bmjopen.bmj.com/site/about/guidelines.xhtml

For peer review only

AUTHOR CONTRIBUTIONS

ND, DD, WV, SM, MK, and CH conceived of the initial study design. DN, AH, and DC were involved in data collection and data processing. DN and SL managed the data. SL, DN, and SBM contributed to data analysis. SL, DN, ND, and DD led the writing of the paper. All authors read and approved the final manuscript.

REFERENCES

1. Bassat, Q., et al., *Validity of a minimally invasive autopsy tool for cause of death determination in pediatric deaths in Mozambique: An observational study.* 2017(1549-1676 (Electronic)).
2. Paganelli, C.R., et al., *The evolution of minimally invasive tissue sampling in postmortem examination: a narrative review.* Global Health Action, 2020. **13**(1): p. 1792682.
3. Castillo, P., et al., *Pathological methods applied to the investigation of causes of death in developing countries: minimally invasive autopsy approach.* PloS one, 2015. **10**(6): p. e0132057.
4. Maixenchs, M., et al., *Willingness to Know the Cause of Death and Hypothetical Acceptability of the Minimally Invasive Autopsy in Six Diverse African and Asian Settings: A Mixed Methods Socio-Behavioural Study.* PLoS Medicine, 2016. **13**(11): p. e1002172.
5. Gurley, E.S., et al., *Family and community concerns about post-mortem needle biopsies in a Muslim society.* BMC medical ethics, 2011. **12**(1): p. 10.
6. Maixenchs, M., et al., *Healthcare providers' views and perceptions on post-mortem procedures for cause of death determination in Southern Mozambique.* PLoS Med, 2018. **13**(7).
7. Blevins, J., et al., *Using Participatory Workshops to Assess Alignment or Tension in the Community for Minimally Invasive Tissue Sampling Prior to Start of Child Mortality Surveillance: Lessons from 5 Sites Across the CHAMPS Network.* Clinical Infectious Diseases, 2019. **69**(Suppl 4).
8. Ngwenya, N., et al., *Community Acceptability of Minimally Invasive Autopsy (MIA) in Children Under Five Years of Age in Soweto, South Africa.* Anthropology Southern Africa, 2017. **40**(2): p. 108-121.
9. Feroz, A., et al., *Perceptions of health professionals regarding minimally invasive tissue sampling (MITS) to identify the cause of death in stillbirths and neonates: results from a qualitative study.* Matern Health Neonatol Perinatol, 2019. **5**: p. 17.

For peer review only - http://bmjopen.bmj.com/site/about/guidelines.xhtml

10. Feroz, A., et al., *Perceptions of parents and religious leaders regarding minimal invasive tissue sampling to identify the cause of death in stillbirths and neonates: results from a qualitative study.* Reprod Health, 2019. **10**(16): p. 53.
11. Das, M.K., et al., *Exploring family, community, and healthcare provider perceptions and acceptability for minimal invasive tissue sampling to identify the cause of death in under-five deaths and stillbirths in North India: a qualitative study protocol.* Reproductive Health, 2019. **16**(3).
12. Ben-Sasi, K., et al., *Acceptability of a minimally invasive perinatal/paediatric autopsy: healthcare professionals' views and implications for practice.* Prenatal Diagnosis, 2013. **33**: p. 307–312.
13. Kang, X., et al., *Parental acceptance of minimally invasive fetal and neonatal autopsy compared with conventional autopsy.* Prenat Diag, 2014. **34**(11): p. 1106-10.
14. Lawrence, S., et al., *Hypothetical acceptability of hospital-based post-mortem pediatric minimally invasive tissue sampling in Malawi: The role of complex social relationships.* PLoS ONE, 2021. **16**(2).
15. Bassat, Q., et al., *Development of a post-mortem procedure to reduce the uncertainty regarding causes of death in developing countries.* The Lancet Global health, 2013. **1**(3): p. e125-e126.
16. Berkley JA, et al., *Childhood Acute Illness and Nutrition (CHAIN) Network: A protocol for a multi-site prospective cohort study to identify modifiable risk factors for mortality among acutely ill children in Africa and Asia.* BMJ Open, 2019. **9**(5): p. e028454. PMID:31061058; PMCID:PMC6502050.
17. Hsieh, H.-F. and S.E. Shannon, *Three approaches to qualitative content analysis.* Qualitative Health Research, 2005. **15**(9).
18. Boeije, H.R., *A purposeful approach to the constant comparative method in the analysis of qualitative interviews.* Quality & Quantity, 2002. **36**(4): p. 391-209.
19. Reeves, S., et al., *Why use theories in qualitative research?* BMJ, 2008. **337**(a949).
20. Ewing, V., et al., *Increasing understanding of the relationship between geographic access and gendered decision-making power for treatment-seeking for febrile children in the Chikwawa district of Malawi.* Malar J, 2016. **15**.
21. Das, M., et al., *Perceptions of family, community and religious leaders and acceptability for minimal invasive tissue sampling to identify the cause of death in under-five deaths and stillbirths in North India: a qualitative study.* Reproductive Health, 2021. **18**(1): p. 168.
22. Das, M., et al., *Why parents agree or disagree for minimally invasive tissue sampling (MITS) to identify causes of death in under-five children and stillbirth in North India: a qualitative study.* BMC Pediatr, 2021. **21**(1): p. 513.
23. Desmond, N., A. Prost, and D. Wight, *Managing risk through treatment-seeking in rural north-western Tanzania: Categorising health problems as malaria and nzoka.* Health, Risk, & Society, 2012. **14**(2): p. 149-170.
24. Sookan, T., G. Zihindula, and D. Wassenaar, *Standard of care for social harms in HIV prevention trials: A South African perspective.* Developing World Bioeth, 2019. **20**: p. 194-199.
25. Montgomery, E.T., et al., *Social harms in female-initiated HIV prevention medical research state of the evidence.* AIDS, 2019. **33**(14): p. 2237-2244.

For peer review only - http://bmjopen.bmj.com/site/about/guidelines.xhtml

26. Mfutso-Bengo, J., et al., *Why do individuals agree to enrol in clinical trials? A qualitative study of health research participation in Blantyre, Malawi.* Malawi Medical Journal, 2008. **20**(2): p. 37-41.
27. Klitzman, R., *The Importance of Social, Cultural, and Economic Contexts, and Empirical Research in Examining "Undue Inducement".* Am J Bioethics, 2005. **5**(5): p. 19-21.
28. Hyun, I., *Fair payment or undue inducement?* Nature, 2006. **442**: p. 629-630.
29. Feroz, A., et al., *A comparison of MITS counseling and informed consent processes in Pakistan, India, Bangladesh, Kenya, and Ethiopia.* Reproductive Health, 2020. **17**(120).
30. Beck, A.M. and C. Konner, A., *Ethical issues in the study of bereavement: The opinions of bereaved adults.* Death Studies, 2007. **31**: p. 783-799.
31. Whitfield, V., et al., *The complexities of recruiting bereaved family members into a research study in the critical care environment: A discussion paper.* Australian Critical Care, 2015. **28**: p. 77-81.

For peer review only

For peer review only - http://bmjopen.bmj.com/site/about/guidelines.xhtml

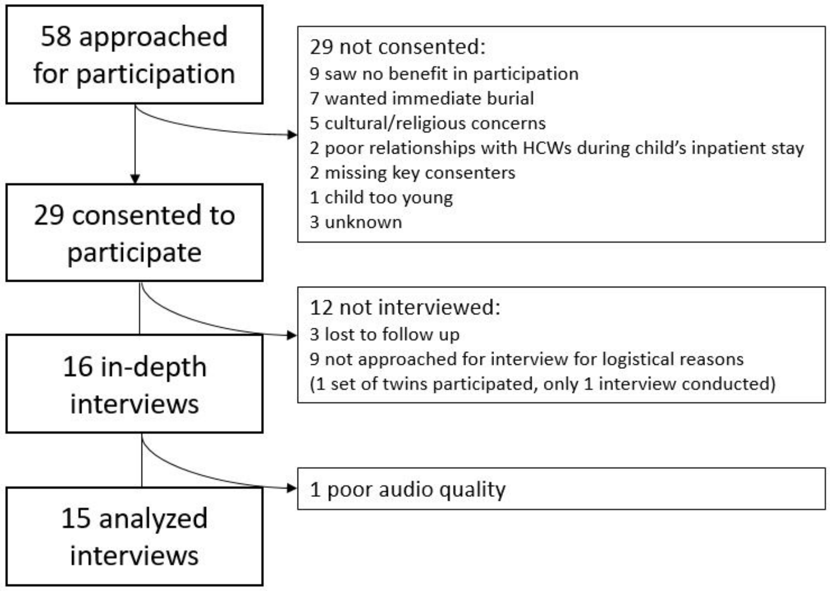

Figure 1. Consent to MITS and interviews.

235x160mm (96 x 96 DPI)

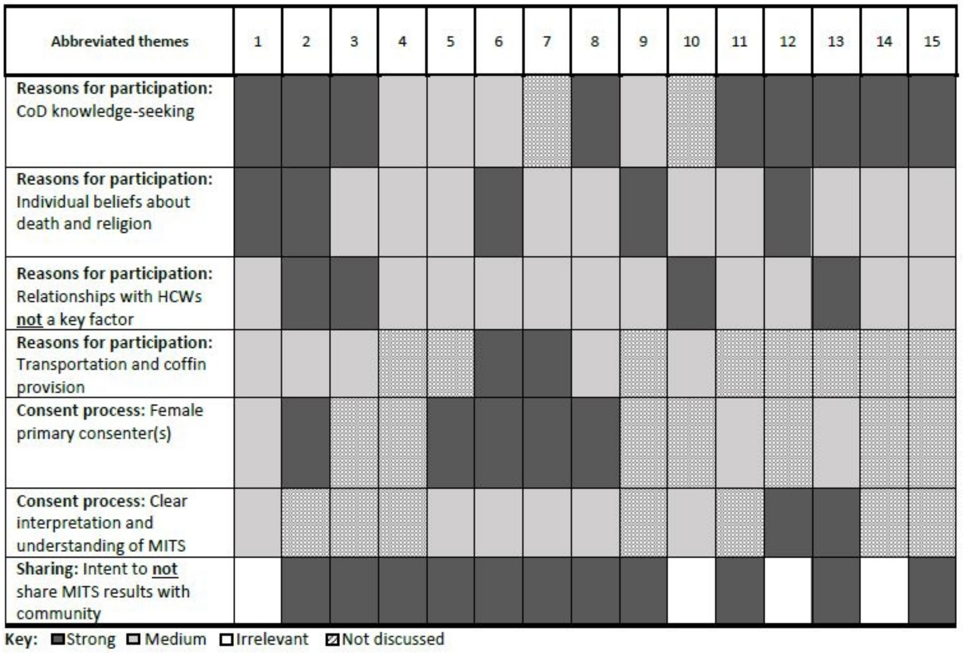

Figure 2. Strength of themes by family.

207x142mm (96 x 96 DPI)

Supplemental Table 1. Interview summaries.

Family #	Summary	Primary caregiver(s)	Caregiver(s) religion	Understood diagnosis available	Primary consenter(s)	Interview participant(s)
**1**	Family first went to multiple health centers, where they reported being poorly treated by HCWs. They enrolled in CHAIN in hopes their engagement in the study would improve the child's health. Family very pleased with care by CHAIN team generally. MITS participation was motivated by wanting to better understand child's CoD but did not understand MITS consent procedures well.	Mother and father	Pentecostal	Yes	Mother and father	Mother and father
**2**	Child's mother reported not getting along with the father. She had previously enrolled in malaria research and enrolled the child in CHAIN. Mother was pleased with CHAIN care but displeased with HCWs at night. She was motivated to know her child's CoD, especially if hereditary.	Mother	Pentecostal	No	Mother (grandmother present for support only)	Mother
**3**	Child's mother was displeased with care provided when child's condition was deteriorating. She consented independently to CHAIN but did not participate in the MITS consent process, deferring to tradition. Child's father and grandfather wanted to know CoD, thinking clinical diagnosis might not be the "real" CoD.	Mother	Protestant	Yes	Father and maternal uncle	Mother
**4**	Child was receiving care at a health center for a month before admission to QECH. Child was enrolled in CHAIN. Family was interested in learning CoD and did not want to take the time to solicit input from other family members during consent process.	Mother and father	Pentecostal	No	Father	Mother and father
**5**	Family did not want to seek care at local health center because they believed care quality there to be poor. Child was enrolled in CHAIN. Child's father had trouble visiting during admission because they lacked funds for transport. He was not involved in consent process. Child's maternal aunt was involved in the MITS consent and initially expressed concerns about organ harvesting. The mother wanted to know CoD.	Mother and father	Pentecostal	No	Mother, maternal aunt and cousin, and cousin's husband	Mother

For peer review only

**6**	Child's father left mother after family tested HIV positive and accused mother of sex work. He visited during child's admission at QECH but was not involved in the consent process. Child was enrolled in a nutrition substudy of CHAIN and mother felt supported by HCWs at QECH. Child's mother was interested in better understanding child's CoD but was primarily motivated by transport and coffin provision, as told to her by staff on the wards before the consent process began.	Mother	Catholic	Yes	Mother	Mother
**7**	Child was enrolled in a nutrition substudy of CHAIN. Family was influenced by transport provision in their decision to consent. Mother consented alone before the child's uncle arrived and he confirmed to proceed.	Mother	Catholic	No	Mother and grandmother	Mother, grandmother, and uncle
**8**	Child's mother was young (last year of secondary school) and initially hid her pregnancy. Child's father recently had a stroke and was receiving treatment. Family was very upset with care, especially lack of testing they were told was going to be done. Family's motivation to participate in MITS was to provide the CoD results to the father's family if they asked, but only if they asked. All three participants were from different faith groups (Islam, Protestant, and Catholic) but raised as Muslim.	Mother	Protestant	Yes	Mother, grandmother, and maternal aunt	Mother, grandmother, and maternal aunt
**9**	Child had sustained severe burns that were being managed on an outpatient basis prior to the last hospital admission. The father's family's church prohibits hospital treatment. However, family elects to seek treatment when needed and accepts church reprimands. While the mother was not involved in MITS consent process (she had returned home), she reported she would have consented as well.	Mother and father	Apostolic	Yes	Maternal aunt and her husband, and maternal uncle	Mother and father
**10**	Child's father visited QECH during the hospital stay. Both parents were dissatisfied with the way they were treated at hospital, and they were particularly upset the mother was never asked about child's condition. Father initially consented by phone. He did not seem to have a clear understanding of the MITS procedure. Consent motivated by feeling that caregivers should do as HCWs ask.	Mother and father	Catholic	No	Father	Mother and father

For peer review only

**11**	Family seemed satisfied with communication by HCWs, although limited. Family was keen to know more about CoD because they did not think the child would die as a consequence of pneumonia diagnosis. Child was buried the day after death and family thought they would have received pressure to not consent by the sub-chief if he would have been involved in decision-making.	Mother	Data unavailable	Yes	Father by phone, maternal grandmother, and great aunt	Mother and paternal grandmother
**12 (both twins enrolled in MiM)**	Child's mother was struggling with mental health condition and limited resources during twins' life. She had quit taking antiretroviral medications for HIV infection prior to their deaths at the recommendation of a local "prophet". Twins' father left their mother before their birth. The mother died shortly after twins' deaths and the uncle was interviewed. They had consented to MITS as a family, hoping it would alleviate blame for the twins' mother.	Mother	Apostolic	Data unavailable	Mother, maternal uncle, and maternal grandmother	Uncle
**13**	Child's mother was not satisfied with care received at a nearby public health center and at a private clinic, so she sought care at QECH without a referral. Family was satisfied with the care at QECH. Family consented to MITS to gain CoD information to protect surviving twin.	Mother and father	Pentecostal	No	Mother, father, paternal aunt, and female friend	Mother and father
**14**	Child's mother left child with father at 5 months after he married a second wife. Biological mother stayed with child at QECH, but grandparents (primary caregivers) did not seek mother's input during consent process because she was no longer the guardian. Health treatment was sought at a health center for child's condition prior to admission at QECH. Child was enrolled in a nutrition study. Family consented because they were interested in learning CoD.	Paternal grandparents	Islam	No	Paternal grandparents, (paternal aunt also present)	Paternal grandparents and stepmother
**15**	Child's father visited occasionally during QECH admission. He noted receiving no information from HCWs during visits. Family consented because they were interested in learning CoD and felt it would be helpful to their community, but they had no intention to share the results due to fears of rumors and misconceptions. They were relieved to receive transportation.	Mother and father	Protestant	Data unavailable	Uncle and father	Uncle and father

Abbreviations: CoD, cause of death; HCWs, healthcare workers; QECH, Queen Elizabeth Central Hospital

For peer review only

For peer review only

Appendix A: Discussion Guide

Number	Topic
T01.	**Introduction**<br>• Offering condolences to the family on the loss<br>• Thanking them for granting Consent to MITS as well as for accepting to be interviewed<br>• Briefing them of their rights (and support available) through the course of the interview<br>• Highlighting the essence of the interview with specific comments on the confidentiality of their responses
T02.	**Socio-demographic details**<br>• Verifying details provided at CHAIN recruitment<br>• Asking on additional information
T03.	**Exit Survey**<br>• Administering exit survey
T04.	**History of illness**<br>• Perceived origins/cause of illness<br>- Traditional causes/Medical causes<br>• Specific stages of illness (e.g. stage that made them decide to go to hospital, inform extended family, who decided on action to be taken etc)<br>• Admission into hospital<br>• Recruitment into the CHAIN study<br>- Consenting for tests while in hospital (including decision making around consent)<br>- Relationship with the main CHAIN team<br>• Previous experience of hospital care<br>• Social support in course of illness
T05.	**Hospital care post death**<br>• Explanation of CoD from the hospital<br>• Understanding of explained CoD<br>- Sufficiency of explained CoD<br>- Alignment with their initial thoughts on CoD<br>• Explained CoD ability to address concerns
T06.	**Approach for MITS**<br>• Consent for MITS<br>• Information given for MITS<br>• Reasons for Consenting to MITS

For peer review only - http://bmjopen.bmj.com/site/about/guidelines.xhtml

	• Decision making
T07.	**MITS and burial practices**
	• Religious and traditional customs observed in child death and burial<br>- Usual burial rites for child (age specific in relation to tradition/religion)<br>• MITS implication on observing customs
T08.	**Sharing of MITS information**
	• People told about the procedure and reasons for telling them<br>• Reactions of the people told, things heard in hospital or community<br>• Family and HH response to the reactions<br>-
T09.	**Knowledge production**
	• Knowledge gained after participating in MITS<br>• Impact of MITS in relating to hospital/HCWs<br>• Impact of MITS in future home care and care-seeking for illnesses<br>• Overall benefits from the procedure/experience
T10.	**Concluding remarks**
	• Regrets<br>• Recommendations for MITS consent approach<br>• Recommendations on releasing results of MITS to families<br>• Likelihood of recommending MITS<br>• General comments

For peer review only

COREQ (COnsolidated criteria for REporting Qualitative research) Checklist

A checklist of items that should be included in reports of qualitative research. You must report the page number in your manuscript where you consider each of the items listed in this checklist. If you have not included this information, either revise your manuscript accordingly before submitting or note N/A.

Topic	Item No.	Guide Questions/Description	Reported on Page No.
**Domain 1: Research team and reflexivity**			
Personal characteristics			
Interviewer/facilitator	1	Which author/s conducted the interview or focus group?	
Credentials	2	What were the researcher's credentials? E.g. PhD, MD	
Occupation	3	What was their occupation at the time of the study?	
Gender	4	Was the researcher male or female?	
Experience and training	5	What experience or training did the researcher have?	
Relationship with participants			
Relationship established	6	Was a relationship established prior to study commencement?	
Participant knowledge of the interviewer	7	What did the participants know about the researcher? e.g. personal goals, reasons for doing the research	
Interviewer characteristics	8	What characteristics were reported about the inter viewer/facilitator? e.g. Bias, assumptions, reasons and interests in the research topic	
**Domain 2: Study design**			
Theoretical framework			
Methodological orientation and Theory	9	What methodological orientation was stated to underpin the study? e.g. grounded theory, discourse analysis, ethnography, phenomenology, content analysis	
Participant selection			
Sampling	10	How were participants selected? e.g. purposive, convenience, consecutive, snowball	
Method of approach	11	How were participants approached? e.g. face-to-face, telephone, mail, email	
Sample size	12	How many participants were in the study?	
Non-participation	13	How many people refused to participate or dropped out? Reasons?	
Setting			
Setting of data collection	14	Where was the data collected? e.g. home, clinic, workplace	
Presence of non-participants	15	Was anyone else present besides the participants and researchers?	
Description of sample	16	What are the important characteristics of the sample? e.g. demographic data, date	
Data collection			
Interview guide	17	Were questions, prompts, guides provided by the authors? Was it pilot tested?	
Repeat interviews	18	Were repeat inter views carried out? If yes, how many?	
Audio/visual recording	19	Did the research use audio or visual recording to collect the data?	
Field notes	20	Were field notes made during and/or after the inter view or focus group?	
Duration	21	What was the duration of the inter views or focus group?	
Data saturation	22	Was data saturation discussed?	
Transcripts returned	23	Were transcripts returned to participants for comment and/or	

For peer review only

Topic	Item No.	Guide Questions/Description	Reported on Page No.
		correction?	
**Domain 3: analysis and findings**			
Data analysis			
Number of data coders	24	How many data coders coded the data?	
Description of the coding tree	25	Did authors provide a description of the coding tree?	
Derivation of themes	26	Were themes identified in advance or derived from the data?	
Software	27	What software, if applicable, was used to manage the data?	
Participant checking	28	Did participants provide feedback on the findings?	
Reporting			
Quotations presented	29	Were participant quotations presented to illustrate the themes/findings? Was each quotation identified? e.g. participant number	
Data and findings consistent	30	Was there consistency between the data presented and the findings?	
Clarity of major themes	31	Were major themes clearly presented in the findings?	
Clarity of minor themes	32	Is there a description of diverse cases or discussion of minor themes?	

Developed from: Tong A, Sainsbury P, Craig J. Consolidated criteria for reporting qualitative research (COREQ): a 32-item checklist for interviews and focus groups. *International Journal for Quality in Health Care*. 2007. Volume 19, Number 6: pp. 349 – 357

Once you have completed this checklist, please save a copy and upload it as part of your submission. DO NOT include this checklist as part of the main manuscript document. It must be uploaded as a separate file.

For peer review only
